# METTL14‐mediated m6A modification of ZFP14 inhibits clear cell renal cell carcinoma progression via promoting STAT3 ubiquitination

**DOI:** 10.1002/ctm2.70232

**Published:** 2025-02-12

**Authors:** Zhuonan Liu, Tianshui Sun, Zhe Zhang, Chiyuan Piao, Chuize Kong, Xiaotong Zhang

**Affiliations:** ^1^ Department of Urology First Hospital of China Medical University Shenyang City Liaoning Province China; ^2^ Department of Obstetrics and Gynecology Shengjing Hospital of China Medical University Shenyang Liaoning China

**Keywords:** ccRCC, m6A, METTL14, STAT3, ubiquitination, ZFP14

## Abstract

Therapeutic options for advanced clear cell renal cell carcinoma (ccRCC) are currently inadequate. Earlier research has shown that the enzyme methyltransferase‐like 14 (METTL14) can suppress ccRCC development through the modification of N6‐methyladenosine (m6A). This study further explored its complex biological functions and underlying molecular mechanisms. Here, we identified zinc finger protein 14 (ZFP14) as a novel target of METTL14‐mediated m6A, and its under‐expression was associated with ccRCC tumourigenesis and progression. Detailed investigations revealed that METTL14 interacted directly with the 3′ untranslated region of ZFP14 mRNA, promoting m6A modification at two specific sites. These modifications were recognised by the protein insulin‐like growth factor 2 mRNA‐binding protein 2 (IGF2BP2), which stabilised and enhanced the expression of ZFP14 mRNA. Functionally, the METTL14/ZFP14 axis suppressed in vitro growth, migration and invasiveness and in vivo proliferation and metastasis of ccRCC cells. ZFP14 potentially regulated numbers of transcripts, among which matrix metalloproteinase 1/3 (MMP1/3) were validated to be under‐expressed by ZFP14. Crucially, ZFP14 interacted with the signal transducer and activator of transcription 3 (STAT3), augmenting its K48‐linked ubiquitination and destabilising it via the proteasome pathway. Moreover, ZFP14 repressed ccRCC cell in vivo growth and metastasis as well as decreasing MMP1/3 levels by under‐expressing STAT3. These observations confirmed that ZFP14 served as both a novel target for METTL14‐mediated m6A modification and a significant tumour suppressor in ccRCC, shedding light on the cellular and molecular operations in ccRCC and opening up possibilities for novel therapeutic strategies.

**Key points:**

- ZFP14 under‐expression is associated with ccRCC tumourigenesis and progression.
- METTL14‐mediated m6A enhances ZFP14 mRNA stability and expression with IGF2BP2 as the reader in ccRCC.
- ZFP14 promotes the degradation of STAT3 by enhancing its K48‐linked ubiquitination, inhibiting ccRCC progression.

## Graphical abstract

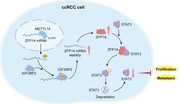

## 1 INTRODUCTION

Renal cell carcinoma (RCC), a leading cause of death among urological cancers, comprises about 4% of all new cancer diagnoses and is responsible for over 170 000 deaths globally each year.^1^, ^2^, ^3^ Clear cell RCC (ccRCC), the most invasive subtype, accounts for more than 80% of all RCC cases and is notorious for its high relapse rates.^4^, ^5^ Often presenting atypical symptoms in its early stages, ccRCC frequently manifests tumour metastasis in over 25% of cases at diagnosis, rendering surgery less feasible and reducing the 5‐year survival rate to below 15%.^6^ Characteristically unresponsive to chemotherapy and radiotherapy, ccRCC presents significant challenges for non‐surgical interventions as the disease progresses.^7^ Despite advancements in targeted drugs and immunotherapy over the past decade, ccRCC treatments often result in low objective response rates and drug tolerance, leading to poor long‐term outcomes.^7^, ^8^ Understanding the molecular underpinnings of ccRCC is imperative to develop more effective diagnostic tools and treatments.

Currently, researchers have identified more than 150 types of internal RNA modifications, with N6‐methyladenosine (m6A) being the most common.^9^ This modification is performed by a group of enzymes known as ‘writers’, which primarily includes methyltransferase‐like 13 (METTL3), METTL14 as well as Wilms tumour 1‐associated protein, along with several other methyltransferases and their cofactors. Conversely, enzymes such as the obesity‐associated protein and the alkylation repair homolog protein 5 (ALKBH5) act as ‘erasers’ to reverse these modifications. The ‘readers’ of m6A, mainly including the YTH domain family proteins and insulin‐like growth factor 2 mRNA‐binding protein (IGF2BP) proteins, determine the fate of m6A‐modified mRNAs, influencing their splicing, localisation, maturation, degradation, stability and translation.^10^ Dysregulation of m6A modification has been implicated as a key factor in various diseases, particularly cancers.^11^ In a 2021 study, it was shown that METTL14, under‐expressed in ccRCC, impedes tumour spread by modulating BPTF expression through the m6A‐dependent mechanism.^12^ Further supporting these findings, our research group verified the diminished levels of METTL14 and reduced m6A enrichment in ccRCC tissues, highlighting how METTL14‐mediated m6A modification adversely regulates ITGB4 expression, a crucial factor in facilitating ccRCC metastasis.^13^ However, the comprehensive understanding of how this epitranscriptomic form influences ccRCC development through multiple biological behaviours remains limited; furthermore, we hypothesise that METTL14 exerts its significant biological functions in ccRCC by targeting additional downstream targets through various mechanisms.

In this study, we identified zinc finger protein 14 (ZFP14), a member of the ZFP family and part of the MDM2 E3 ubiquitin ligase complex, as a new target of METTL14‐mediated m6A.^14^ To date, research on ZFP14 is sparse, and its role in ccRCC has not been explored. Our research indicated that ZFP14 functioned as a tumour suppressor in ccRCC by promoting the ubiquitination of signal transducer and activator of transcription 3 (STAT3), an established oncogene implicated in multiple cancers, including ccRCC.^15^ Overall, our research provides deeper insights into m6A modification in ccRCC and offers potential strategies for addressing this neoplasm, with a systematic and comprehensive exploration of related molecular mechanisms.

## 2 MATERIALS AND METHODS

### 2.1 Bioinformatic analyses of public data

To conduct bioinformatic studies, data from 523 tumour samples and 72 normal kidney samples were extracted from ccRCC patients enrolled in The Cancer Genome Atlas (TCGA) database. Results were presented using websites UALCAN and GEPIA. The URLs are shown in the corresponding figure legends.

### 2.2 Patient samples

Patients with ccRCC who had their kidneys removed for treatment provided the samples. We utilised 50 pairs of ccRCC tissues and corresponding adjacent normal kidney tissues for the quantitative real‐time PCR (qRT‐PCR) assay, 24 pairs for the western blot assay and the 24 pairs for the total m6A quantification assay. All participants provided written informed consent, and the Research Ethics Committee of China Medical University given their approval (NO. 2022‐48‐2). An environment of −80°C was used to preserve the samples.

### 2.3 Cell lines and cell culture

The human embryonic kidney 293T cells, human ccRCC cell lines 786‐O, 769‐P, OS‐RC‐2, Caki‐1 and ACHN were procured from the Chinese Academy of Sciences Type Culture Collection Cell Bank in Shanghai, China, and were cultured at 37°C with 5% CO_2_. The Caki‐1, ACHN, 786‐O, 769‐P, OS‐RC‐2 and HEK293T cells were cultured in McCoy's 5A medium, MEM medium and RPMI medium from Hyclone (Beijing, China), respectively. Each medium contained 10% foetal bovine serum (FBS) purchased from Biological Industries (Beit‐HaEmek, Israel).

### 2.4 Small interfering RNA transfection

JTS‐BIO Co. (Wuhan, China) provided the small interfering RNA (siRNA) oligonucleotides that were used to target METTL14, ZFP14, IGF2BP1, IGF2BP2 and IGF2BP3. We followed the manufacturer's instructions when transfecting with LipofectamineTM 3000 (Invitrogen, Thermo Fisher Scientific, Waltham, USA). The siRNA sequences can be found in Table S3.

### 2.5 Plasmid construction and cell transfection

Overexpression plasmids (Flag‐tagged METTL14 and IGF2BP2) and short‐hairpin RNA (shRNA) oligonucleotides (sh‐METTL14 and sh‐IGF2BP2), along with their corresponding negative controls (NCs), were packaged into lentiviral particles by GeneChem (Shanghai, China). Lentiviral transfection followed protocols provided by GeneChem. Plasmids for ZFP14, Flag‐tagged ZFP14, STAT3, His‐tagged STAT3, HA‐tagged ubiquitin and dual‐luciferase reporters were also purchased from GeneChem and transfected using Lipofectamine™3000 (Invitrogen, Thermo Fisher Scientific). The sequences of shRNAs are listed in Table S4.

### 2.6 Quantitative real‐time PCR

For total RNA extraction and reverse transcription, we followed the manufacturer's recommendations and utilised RNAiso Plus and Prime Script RT Master Mix from Takara Biotechnology in Dalian, China, respectively. The Sybr Premix Ex Taq™ kit from Takara Biotechnology was used to conduct qRT‐PCR on a LightCycler™ 480 II equipment from Roche in Basel, Switzerland. The 2^−ΔΔCt^ technique was employed for data analysis. For the clinical samples, the internal reference gene was β‐actin, whereas for the cell line samples, GAPDH was utilised. The primer sequences are provided in Table S5.

### 2.7 Western blot assay

The total protein from cells, clinical tissues and in vivo tumours was extracted using RIPA lysis buffer that contained 1% phenylmethylsulphonyl fluoride (PMSF). The protein concentrations were then measured using a bicinchoninic acid test kit from Beyotime in Nantong, China. Proteins underwent 10% SDS/PAGE separation, were then transferred to PVDF membranes with a 0.2 µm thickness and were blocked with TBST solution containing 5% non‐fat milk. Following an overnight incubation with primary antibodies (Table S6) at 4°C, the membranes were treated for 1 h at 37°C with secondary antibodies conjugated with horseradish peroxidase at a dilution of 1:5000. A chemiluminescence system (Bio‐Rad, California, USA) and an EasySee kit (TransGen Biotech, Beijing, China) were used to visualise protein bands. Using ImageJ software, the intensities of the bands were measured.

### 2.8 Total m6A quantification

We followed the instructions provided by EpiGentek (USA) to measure the overall m6A levels in the samples using a m6A RNA methylation measurement kit. Sourcing and incubation with capture and detection antibodies, enhancer solution, colour development solution and 200 ng of RNA from each sample was the basic procedure. We used the manufacturer‐supplied formula to determine the m6A percentage after measuring the absorbance at 450 nm.

### 2.9 Real‐time cell assay

Cells from each group were dissociated and seeded into E‐plates at a density of 2.0 × 10^3^ cells per well and cultured regularly. Cell indexes were recorded every 5 min for 60 h by an xCELLigence System (Roche), with data automatically exported and analysed in GraphPad Prism 8.0 (GraphPad Software, La Jolla, USA) to generate cell growth curves.

### 2.10 5‐Ethynyl‐2′‐deoxyuridine assay

To measure cell proliferation, we followed the manufacturer's recommendations and used an 5‐ethynyl‐2′‐deoxyuridine (EdU) kit (Beyotime). To summarise, the cells were initially co‐cultured with a working solution (1:1000) at 37°C with 5% CO2. After that, they were fixed with 4% paraformaldehyde for 15 min and treated with 0.3% Triton X‐100 for 30 min, both at room temperature. After that, the cells were left to incubate in the dark at room temperature for 30 min with the click reaction solution, and then for 10 min with the Hoechst solution, all under the same circumstances. A fluorescence microscope (Olympus Corporation, Tokyo, Japan) was utilised to collect fluorescent images, and cell counting was done using ImageJ software.

### 2.11 Wound‐healing assay

In six‐well plates, where the cell density was around 90% confluent, a sterile 1000 µL tip was utilised to make a vertical scratch in the centre of each well. The EVOS XL system, AMEX1200, from Life Technologies Corp. in Bothell, USA, was used to obtain the first picture. A further picture was captured at the same location after the cells had been incubated in FBS‐free media for 48 h. The wound‐healing rate was calculated using ImageJ.

### 2.12 Cell invasion assay

Transwell chambers with 8 µm pore (Corning, USA) were pre‐coated with 25% Matrigel (BD, New Jersey, USA) diluted in FBS‐free medium. Each lower chamber received 600 µL of 2% FBS media, whereas each top chamber received 200 µL of FBS‐free medium containing 3.0 × 10^4^ transfected cells. Cells that remained in the upper chamber were washed out with PBS after 48 h of incubation in the regular cell‐culturing atmosphere, and cells that stuck to the bottom surface were stained with crystal violet. Counting cells was done using ImageJ software after images were taken of stained cells using the aforementioned inverted microscope.

### 2.13 RNA stability assay

At predetermined intervals (0, 3, 6 h), the transfected cells were grown in 10% FBS media that contained 5 µg/mL of actinomycin D (Sigma–Aldrich, USA). The next step was to do qRT‐PCR analysis on the isolated total RNA in preparation for cDNA synthesis. With the help of GraphPad Prism 8.0, RNA degradation curves were created.

### 2.14 Methylated RNA immunoprecipitation qPCR assay

A methylated RNA immunoprecipitation (RIP) (MeRIP) kit from BersinBio in Guangzhou, China, was utilised in accordance with the manufacturer's guidelines. Initially, total RNA was isolated using RNAiso Plus and subjected to chemical fragmentation. One‐tenth of the RNA served as input, while the remainder was incubated with an m6A antibody (ab208577; Abcam, Cambridge, UK). Immunoprecipitated RNA was isolated using A/G magnetic beads. Reverse transcription was then performed on both input and immunoprecipitated RNA, followed by qPCR.

### 2.15 RIP qPCR (RIP‐qPCR) assay

The assay was conducted using an RIP kit (BersinBio) and antibodies for METTL14 (26158‐1‐AP; Proteintech, Wuhan, China), IGF2BP2 antibody (11601‐1‐AP; Proteintech) and IgG (from the RIP kit). A fixed portion of cell lysate was preserved as input, and the rest was incubated with equal amounts of the specified antibodies and A/G magnetic beads for immunoprecipitation. qPCR was performed after reverse transcription of both input and immunoprecipitated RNA.

### 2.16 RNA pulldown assay

Table S7 lists the sequences of the three probes produced by JTS‐BIO Co.: two sense probes, two m6A‐modified probes that mimic the target portions of ZFP14 3′ untranslated region (3′UTR) and a NC probe. We biotinylated all of the probes. An RNA pulldown kit (BersinBio) was applied to perform RNA pulldown assay. Basically, the biotinylated probes were utilised to create RNA secondary structures using. Subsequently, streptavidin magnetic beads were added to the mix. Cell lysates were mixed with the biotinylated RNA‐bead complexes, reserving a portion as input. Beads were collected, and proteins bound were eluted for analysis. Proteins from both the pulldown and input groups were examined by western blot assay.

### 2.17 Dual‐luciferase reporter assay

After 48 h of transfection with ZFP14‐3′UTR luciferase plasmids, cells were harvested from 24‐well plates. Following the directions provided by the manufacturer, the dual‐luciferase assay kit (Promega. Madison, USA) was used for calculation of the activity of Firefly and Renilla luciferases. To determine relative firefly luciferase activities, the equivalent Renilla activities were used as a standard.

### 2.18 MeRIP sequencing and mRNA‐seq

Novogene (Beijing, China) conducted MeRIP sequencing (MeRIP‐seq) on control cells and METTL14‐overexpressed Caki‐1 cells. For the purpose of immunoprecipitation, RNA was first fragmented and then treated with a polyclonal antibody against m6A (Synaptic Systems). Sequencing was carried out on an Illumina NovaSeq platform with a paired‐end read length of 150 bp. The libraries were constructed using immunoprecipitated and input RNAs using the NEBNext Ultra RNA Library Prep Kit for Illumina (New England Biolabs). To find m6A peaks with significant changes, the cutoff values were FDR < 0.05, *p* < .05 and |log2 fold change (FC)| > 1. mRNA‐seq was additionally conducted by Novogene on control cells and Caki‐1 cells over‐expressing ZFP14. Prior to library preparation and sequencing on the Illumina NovaSeq 6000, the total RNA quality and integrity were evaluated using the RNA nano 6000 assay kit on a bioanalyser 2100 system (Agilent Technologies, CA, USA). Differentially expressed gene (DEG) filtration was performed using cutoff values of |log2FC| > 1 and *p* < .05.

### 2.19 Protein stability assay

Cells, either stably transfected or 24 h post‐siRNA transfection, were cultured in media containing 100 µg/mL cycloheximide (Selleck, Inc., Houston, USA) for designated durations. Protein extraction was performed as previously described and so was western blot experiment.

### 2.20 Co‐immunoprecipitation and ubiquitination assay

Cells, transfected or not, were lysed using RIPA buffer containing 1% PMSF and 1% protease inhibitor. In some cases, 10 µM MG132 (Selleck) was added to incubate cells for 5 h before lysis to inhibit proteasomes, as indicated in figures. A fixed proportion of the lysate served as input, while the remainder was incubated with equal amounts of antibody. Antibodies used included STAT3 antibody (10253‐2‐AP; Proteintech), His‐tagged (66005‐1‐lg; Proteintech), Flag‐tagged (66008‐4‐Ig; Proteintech) and IgG (30000‐0‐AP; Proteintech). Immunoprecipitated products were collected with A/G magnetic beads, and both immunoprecipitated and input proteins were analysed by western blot. The ubiquitination assay, based on the Co‐immunoprecipitation (Co‐IP) assay, involved immunoprecipitating endogenous or exogenous STAT3 and detecting its ubiquitination levels via western blot by using 7.5% SDS/PAGE.

### 2.21 Animal experiments

China Medical University's Ethics Committee for the Welfare of Medical Experimental Animals (NO. CMUKT2023265) gave its stamp of approval to all of the animal research. Female BALB/c‐nude mice, 6 weeks old, were procured from Vital River Laboratories in Beijing, China, and kept in a pathogen‐free environment in the Experimental Animal Department at China Medical University. Under the skin of each mouse's right flank, 200 µL of a mixture of FBS‐free media and 40% Matrigel containing 1.0 × 10^6^ transfected ACHN cells was introduced for the tumour growth investigation. Tumours were removed and weighted after 25 days, and the mice were put to sleep. Each mouse's tail vein was injected with 1.0 × 10^5^ ACHN cells in 200 µL of pathogen‐free PBS to create a tail vein‐lung metastasis model for the tumour metastasis study. After a period of 35 days, the lungs were removed from the dead mice, preserved in formalin, and then stained with haematoxylin and eosin (HE) for microscopic examination.

### 2.22 Statistical analyses

Data analysis was performed using GraphPad Prism version 8.0, which facilitated graph creation. Statistical significance between two groups was evaluated via a two‐tailed Student's *t*‐test, with results expressed as mean ± standard deviation (SD). The ANOVA test was employed to assess differences among multiple groups. Pearson correlation analysis was used to determine the relationships between two variables, while survival rates were analysed using the log‐rank test. A *p* value less than .05 was deemed statistically significant.

## 3 RESULTS

### 3.1 ZFP14 is a newly identified target of METTL14‐mediated m6A in ccRCC

According to western blot results, endogenous METTL14 was found at its lowest levels in Caki‐1 cells among the five ccRCC cell lines, whereas ACHN cells exhibited the highest levels (Figure S1A). Consequently, Caki‐1 cells over‐expressing this m6A writer and those transfected with the empty vector were established for MeRIP‐seq, aimed at identifying potential target genes of METTL14‐mediated m6A modification (Figure S1B). The results revealed a high enrichment of the m6A consensus motif ‘GGACU’ in Caki‐1 cells, regardless of METTL14 over‐expression (Figure S1C). Among 114 genes showing significantly increased m6A levels following METTL14 over‐expression, ZFP14 displayed the highest increase in m6A abundance, located on the 3′UTR of its mRNA (Figure S1D and Table S1). Through nucleotide sequence analysis, two segments containing the motif were identified within the target region of ZFP14's 3′UTR, as confirmed by sequencing results (Figure S1E). Recognising multiple and important biology functions of the ZFP14 family, we hypothesised that ZFP14 participated in ccRCC tumourigenesis or progression, regulated by METTL14‐mediated m6A modification.

### 3.2 Under‐expression of ZFP14 is associated with ccRCC tumourigenesis and progression

To confirm ZFP14 as a potential regulator of ccRCC tumourigenesis or progression, we conducted bioinformatic analyses using the TCGA public database, which provided relevant transcriptome data. Results indicated that, compared with non‐paired normal samples, ZFP14 was generally under‐expressed in ccRCC samples, although not statistically significant (Figure S2A). Nonetheless, lower levels of ZFP14 transcripts were associated with poorer overall and disease‐free prognosis in ccRCC patients (Figure S2B,C). Additionally, ZFP14 expression decreased with increasing tumour stage, pathological grade and the presence of lymph node metastasis (Figure S2D–F). Based on these findings, we considered ZFP14 a potential tumour suppressor in ccRCC.

**FIGURE 1:**
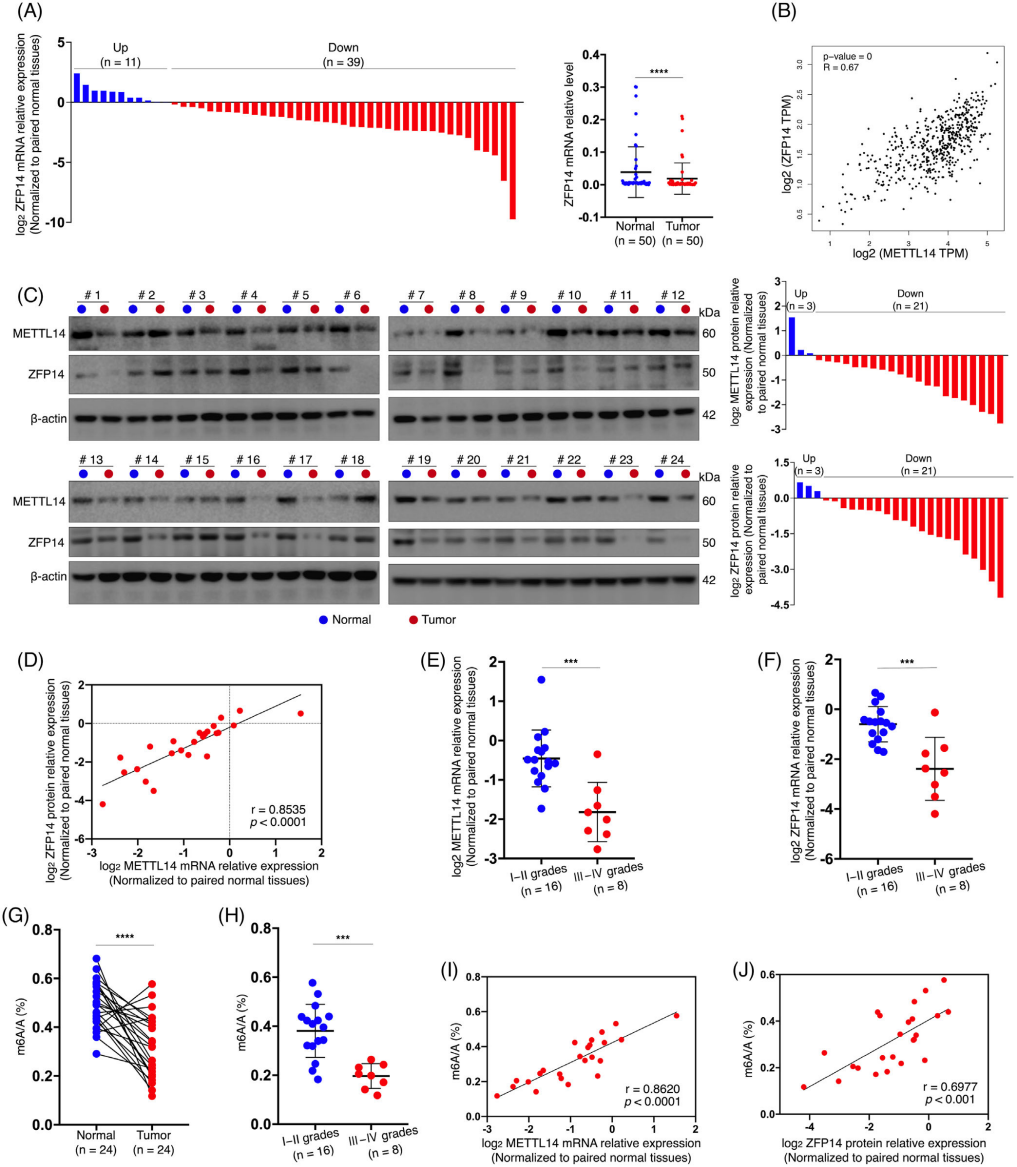
Under‐expression of ZFP14 is associated with ccRCC tumourigenesis and progression. (A) ZFP14 mRNA abundance in 50 pairs of clinical samples detected by qRT‐PCR. (B) Expression correlation between ZFP14 and METTL14 mRNA in TCGA database. (C) ZFP14 protein abundance in 24 pairs of clinical samples detected by western blot assay. (D) Correlation between ZFP14 and METTL14 protein levels according to the western blot analysis. (E, F) METTL14 (E) and ZFP14 (F) protein levels in the ccRCC samples of low and high pathological grades. (G) Total m6A abundance in the 24 pairs of clinical samples detected by total m6A quantification assay. (H) Total m6A abundance in the ccRCC samples of low and high pathological grades. (I, J) Correlation of total m6A abundance with METTL14 (I) and ZFP14 (J) protein levels in the 24 ccRCC samples. Student's *t*‐test was used to analyse the difference between two groups. Correlations between two indices were calculated using Pearson correlation analysis. ***p < .001 and ****p < .0001.

Subsequently, a qRT‐PCR experiment was performed on 50 pairs of clinical samples, revealing significantly reduced ZFP14 mRNA levels in ccRCC tissues compared with adjacent normal kidney tissues (Figure 1A). Moreover, bioinformatic analysis showed a positive correlation between ZFP14 and METTL14 transcript levels (Figure 1B). A western blot assay on 24 pairs of tissues not only confirmed the reduced abundance of METTL14 and ZFP14 proteins in most ccRCC cases but also demonstrated a positive correlation between the expressions of these two proteins (Figure 1C,D). More importantly, ccRCC of higher pathological grades exhibited lower expressions of METTL14 and ZFP14 than those of lower grades (Figure 1E,F). Furthermore, a total m6A quantification assay conducted on the same 24 pairs of samples revealed a significant decrease in m6A levels in tumour tissues, particularly in those of higher grades (Figure 1G,H). Additionally, total m6A richness in the tumours was positively correlated with the expressions of METTL14 and ZFP14 (Figure 1I,J). Taken together, these findings suggest that the diminished expression of ZFP14 is associated with ccRCC tumourigenesis and progression, likely due to reduced m6A modification mediated by METTL14.

### 3.3 METTL14‐mediated m6A modification enhances ZFP14 expression

**FIGURE 2:**
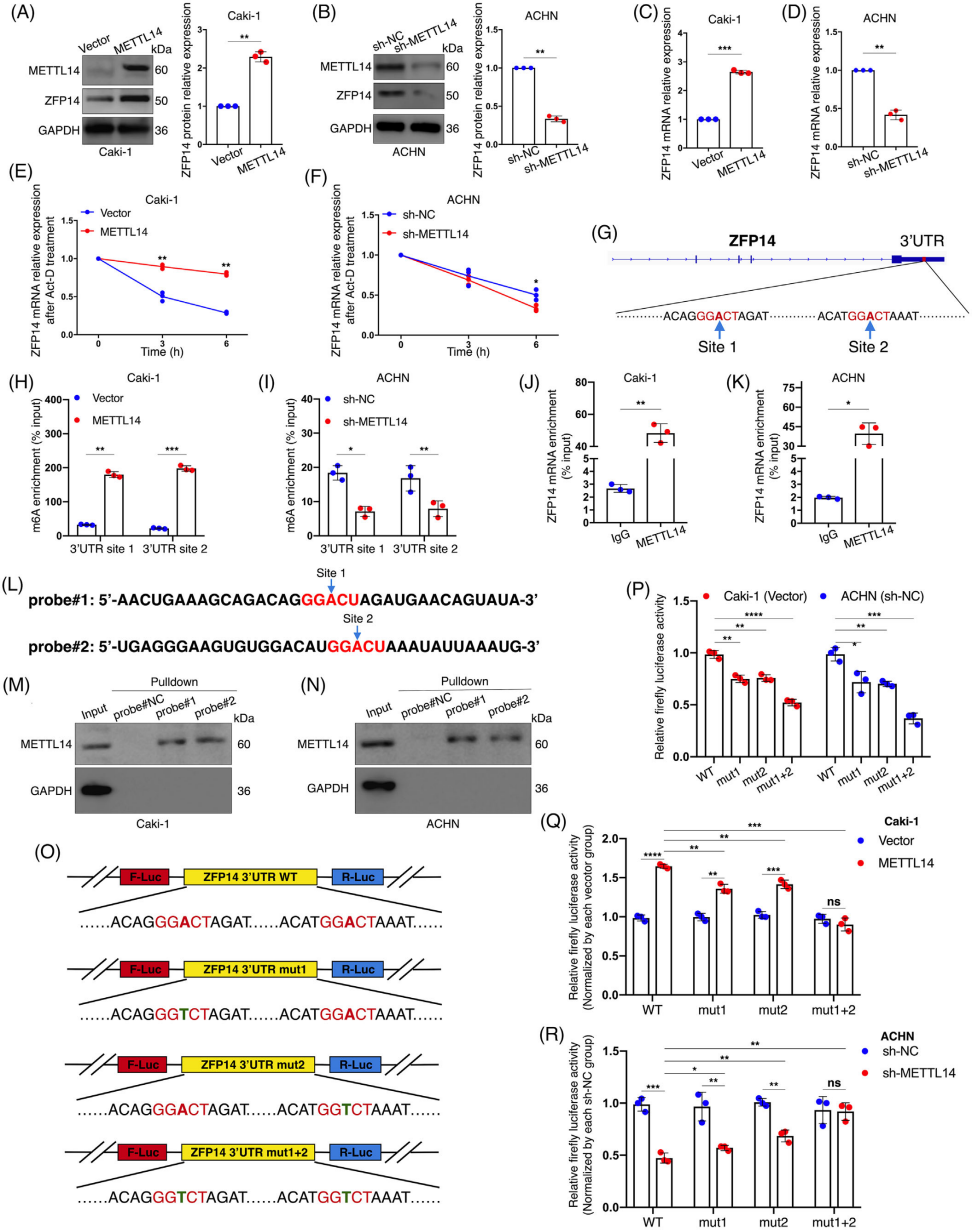
METTL14‐mediated m6A modification enhances ZFP14 expression. (A and B) Alterations of METTL14 and ZFP14 protein levels after METTL14 over‐expression (A) and METTL14 knockdown (B) in the indicated cells detected by western blot assay. (C and D) Alterations of ZFP14 mRNA levels after METTL14 over‐expression (C) and METTL14 knockdown (D) in the indicated cells detected by qRT‐PCR assay. (E, F) Alterations of ZFP14 mRNA degrading velocity after METTL14 over‐expression (E) and METTL14 knockdown (F) in the indicated cells detected by RNA stability assay. (G) Schematic representation of positions of m6A motifs within ZFP14 3′UTR. (H and I) Alterations of m6A enrichment on the two regions of ZFP14 3′UTR followed by METTL14 over‐expression (H) and METTL14 knockdown (I) in the indicated cells detected by MeRIP‐qPCR assay. (J and K) Interactions between METTL14 and ZFP14 mRNA in Caki‐1 (J) and ACHN (K) cells detected by RIP‐qPCR assay. (L) Sequences of biotinylated RNA probes targeting ZFP14 3′UTR. (M and N) Interactions between METTL14 and the indicated RNA probes in Caki‐1 (M) and ACHN (N) cells. (O) Schematic representation of the construction of dual‐luciferase plasmids containing the wild‐type and mutated ZFP14 3′UTR. (P–R) Relative luciferase activities detected after the indicated dual‐luciferase plasmids were transfected into METTL14‐overexpressing Caki‐1 cells, METTL14‐silencing ACHN cells and the corresponding negative control cells. Alterations of relative luciferase activities in the negative control cells followed by ZFP14 3′UTR mutations (P). Alterations of relative luciferase activities respectively led by METTL14 over‐expression (Q) and METTL14‐underexpression (R) with or without ZFP14 3′UTR mutations. Data are presented as means ± SD based on triple independent experiments. Student's *t*‐test was used for analyses. *p < .05, **p < .01, ***p < .001 and ****p < .0001. ns, non‐significant.

Previously recognised as a tumour suppressor, METTL14's mechanisms remain inadequately understood despite prior studies.^12^, ^13^ Based on the MeRIP‐seq results and the previously identified abnormal abundance of ZFP14 in ccRCC, experiments were conducted to confirm whether ZFP14 is a downstream target of METTL14‐dominated m6A, potentially responsible for its impaired expression. Western blot analysis showed that ZFP14 protein was enriched in Caki‐1 cells over‐expressing METTL14, while its abundance was reduced in ACHN cells with stable or transient knockdown of this m6A writer (Figures 2A,B and S3). Similarly, the positive regulatory effect of METTL14 on ZFP14 mRNA expression was confirmed by qRT‐PCR (Figure 2C,D). These findings aligned with correlation analyses, suggesting ZFP14 as a downstream target of METTL14, irrespective of m6A regulation. Given the impact of METTL14 on ZFP14 expression at the transcript level, it was speculated that m6A mediated by METTL14 influences the stability of ZFP14 mRNA, reflecting common knowledge of how this epitranscriptomic modification affects RNA fate.^10^ An RNA decay assay revealed slower degradation of ZFP14 mRNA when METTL14 was over‐expressed, whereas METTL14 knockdown resulted in decreased transcript stability of ZFP14 (Figure 2E,F).

The MeRIP‐seq results identified a concentration of m6A within the ZFP14 3′UTR, a region typically governing RNA decay.^16^ Within this region, two ‘GGACU’‐contained adenosines were pinpointed as potential N6‐methylated sites, named site1 and site 2 (Figure 2G). PCR primers targeting each region of the sites were synthesised for MeRIP‐qPCR assay. Results showed that METTL14 over‐expression increased m6A enrichment at both regions, whereas METTL14 knockdown in ACHN cells produced the opposite effect (Figure 2H,I). The direct interaction between METTL14 protein and ZFP14 mRNA was further confirmed in ccRCC cell lines via RIP‐qPCR (Figure 2J,K). To ascertain whether METTL14 regulated ZFP14 expression by targeting these m6A sites, an RNA pulldown assay was first performed using biotin‐labelled single‐stranded RNA probes that mimic the two regions (Figure 2L). The interaction of these probes with METTL14 was observed (Figure 2M,N). A dual‐luciferase reporter assay was then conducted with luciferase reporter vectors containing either the wild‐type ZFP14 3′UTR or three mutant types (Figure 2O). Results indicated that, in the NC cell lines, mutations at sites 1 and 2 decreased luciferase activity, with dual mutations causing the most substantial reduction (Figure 2P). Further analysis showed an increase in luciferase activity of the constructs harbouring the wild‐typed ZFP14 3′UTR following METTL14 over‐expression, which was reduced by mutations at either m6A site and abolished by the dual mutations (Figure 2Q). Conversely, METTL14 silencing in ACHN cells reduced expression from the normal ZFP14 3′UTR, a phenomenon reversible by the double mutations (Figure 2R).

In summary, METTL14 directly functions on the 3′UTR of ZFP14 mRNA, enhancing its m6A modifications, which leads to increased transcript stability and subsequently, ZFP14 over‐expression.

### 3.4 IGF2BP2 participates in METTL14‐mediated m6A regulation of ZFP14 expression

**FIGURE 3:**
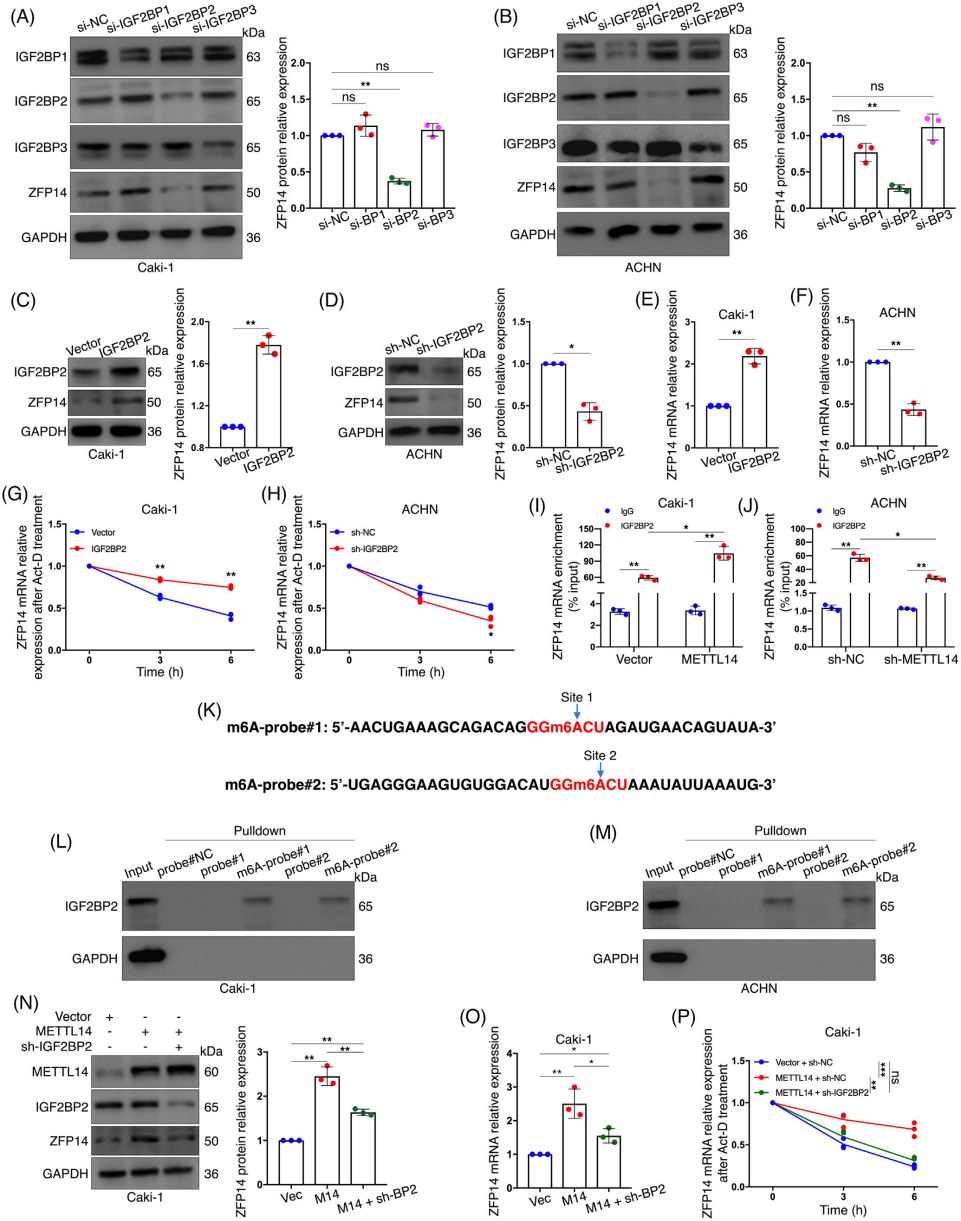
IGF2BP2 participates in METTL14‐mediated m6A regulation of ZFP14. (A and B) Alterations of IGF2BP1/2/3 and ZFP14 protein levels in Caki‐1 (A) and ACHN (B) cells after the indicated treatments. (C and D) Alterations of IGF2BP2 and ZFP14 protein levels after IGF2BP2 over‐expression (C) and IGF2BP2 knockdown (D) in the indicated cells detected by western blot assay. (E and F) Alterations of ZFP14 mRNA levels after IGF2BP2 over‐expression (E) and IGF2BP2 knockdown (F) in the indicated cells detected by qRT‐PCR assay. (G and H) Alterations of ZFP14 mRNA degrading velocity after IGF2BP2 over‐expression (G) and IGF2BP2 knockdown (H) in the indicated cells detected by RNA stability assay. (I and J) Alterations of the interaction between IGF2BP2 and ZFP14 mRNA followed by METTL14 over‐expression (I) and METTL14 knockdown (J) detected by RIP‐qPCR assay. (K) Sequences of biotinylated RNA probes targeting ZFP14 3′UTR with m6A modification. (L and M) Interactions between METTL14 and the indicated RNA probes in Caki‐1 (L) and ACHN (M) cells. (N) Alterations of METTL14, IGF2BP2 and ZFP14 protein levels detected by western assay after the indicated treatments. (O) Alterations of ZFP14 mRNA levels detected by qRT‐PCR assay after the indicated treatments. (P) Alterations of ZFP14 mRNA degrading velocities detected by RNA stability assay after the indicated treatments. Data are presented as means ± SD based on three independent experiments. Student's *t*‐test was used for analyses. *p < .05, **p < .01and ***p < .001. ns, non‐significant.

We further investigated the m6A reader involved in the regulation of ZFP14 expression. The IGF2BPs are known to enhance the stability of target mRNAs upon recognising m6A.^11^ Initially, IGF2BP1, 2 and 3 were respectively knocked down in Caki‐1 and ACHN cells. Western blot results showed that ZFP14 protein level was reduced only in the IGF2BP2‐silenced group, identifying this reader as a potential effector (Figure 3A,B). Besides, we also observed a higher efficiency for IGF2BP2 knockdown in ACHN cells. Consequently, IGF2BP2 was over‐expressed in Caki‐1 cells, resulting in increased ZFP14 protein abundance; conversely, a reduction was observed following the stable knockdown of this reader (Figure 3C,D). IGF2BP2's stimulative effect on ZFP14 mRNA expression was confirmed via qRT‐PCR (Figure 3E,F). RNA stability assay was then conducted after either over‐expression or knockdown of IGF2BP2 to verify whether this reader enhanced the stability of ZFP14 transcripts, yielding positive results (Figure 3G,H). Using RIP‐qPCR assay, we confirmed the direct interaction between IGF2BP2 and ZFP14 mRNA in both Caki‐1 and ACHN cells; additionally, METTL14 over‐expression increased this interaction, while its reduction had the opposite effect, supporting the notion that IGF2BP2 influences ZFP14 transcripts in an METTL14‐mediated m6A‐dependent manner (Figure 3I,J). On the base of the RNA probes above, two m6A‐modified RNA probes were synthesised for RNA pulldown experiment to further investigate this interaction (Figure 3K). Results demonstrated that IGF2BP2 directly interacted with the two fragments of the ZFP14 3′UTR only when the target adenosines were N6‐methylated (Figures 3L,M). To further confirm IGF2BP2's role in METTL14's regulation of ZFP14 expression, we silenced IGF2BP2 following METTL14 enrichment in Caki‐1 cells. It was observed that with lessened IGF2BP2, the increase in ZFP14 protein and mRNA levels following METTL14 over‐expression was reversed (Figures 3N,O). Additionally, a similar rescuing effect on ZFP14 mRNA stability was observed (Figure 3P).

In conclusion, the reader IGF2BP2 interacts with the 3′UTR of ZFP14 mRNA modified by METTL14‐mediated m6A, leading to enhanced stability and expression of the transcript.

### 3.5 ZFP14 is required for METTL14's inhibitory effect on ccRCC progression

**FIGURE 4:**
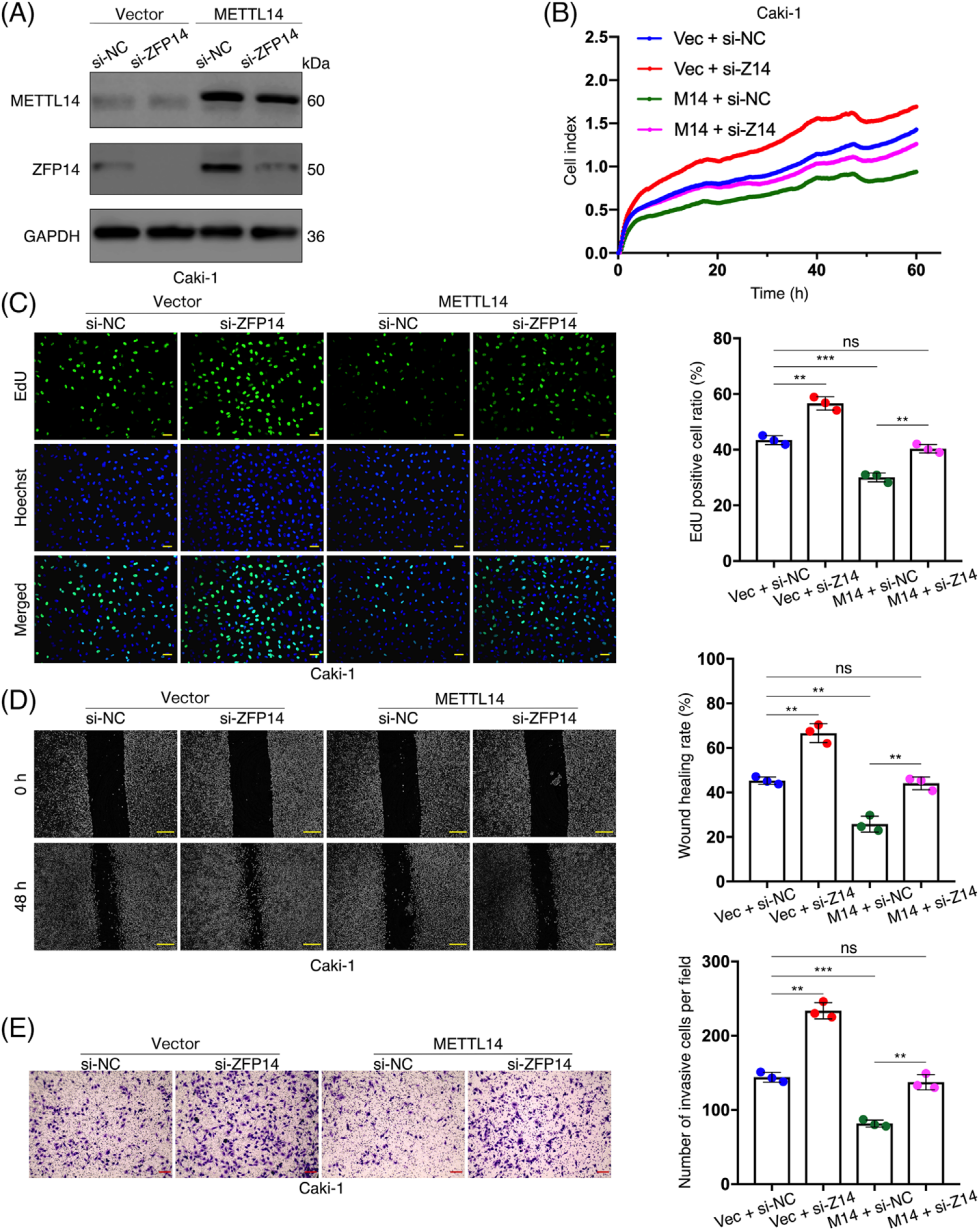
METTL14/ZFP14 axis inhibits in vitro proliferation, migration and invasion of ccRCC cells. (A) METTL14 and ZFP14 protein levels in Caki‐1 cells of the indicated disposals detected by western blot assay. (B) Proliferation curves of Caki‐1 cells of the indicated disposals detected by RTCA assay. (C) Proliferation status of Caki‐1 cells of the indicated disposals detected by EdU assay. Bar scale = 20 µm. (D) Migratory rates of Caki‐1 cells of the indicated disposals detected by wound‐healing assay. Bar scale = 50 µm. (E) Invasiveness of Caki‐1 cells of the indicated disposals detected by cell invasion assay. Bar scale = 50 µm. Data are presented as means ± SD from three independent experiments. Student's *t*‐test was used for analyses. **p < .01, ***p < .001. ns, non‐significant.

We sought to explore the biological functions of ZFP14 in ccRCC, particularly its role in METTL14's tumour‐suppressing effects, as previously demonstrated.^13^, ^17^ Initially, two strands of siRNA were synthesised to silence ZFP14, with the first being selected for subsequent studies due to its effectiveness (Figure S4). In our experiments, ZFP14 was suppressed in Caki‐1 cells, whether they over‐expressed METTL14 or carried the empty vector. Conversely, ZFP14 was up‐regulated in ACHN cells, with or without METTL14 inhibition (Figures 4A and S5A). Subsequent in vitro rescue assays demonstrated that decreased ZFP14 expression stimulated Caki‐1 cell proliferation and counteracted the growth inhibitory effect of METTL14 over‐expression (Figure 4B). Furthermore, the proliferation of ACHN cells was inhibited by ZFP14 over‐expression that also effectively countered the enhanced cell growth induced by silenced METTL14 (Figure S5B). This dependence of METTL14's role in inhibiting ccRCC cell proliferation on ZFP14 was further confirmed by the EdU assay, which measured cell growth rates by detecting DNA replication activity (Figures 4C and S5C). Similarly, the inhibitory effects of the METTL14/ZFP14 axis on the migratory capacity and invasiveness of ccRCC cells *were* demonstrated through wound‐healing and cell invasion assays (Figures 4D,E and S5D,E).

**FIGURE 5:**
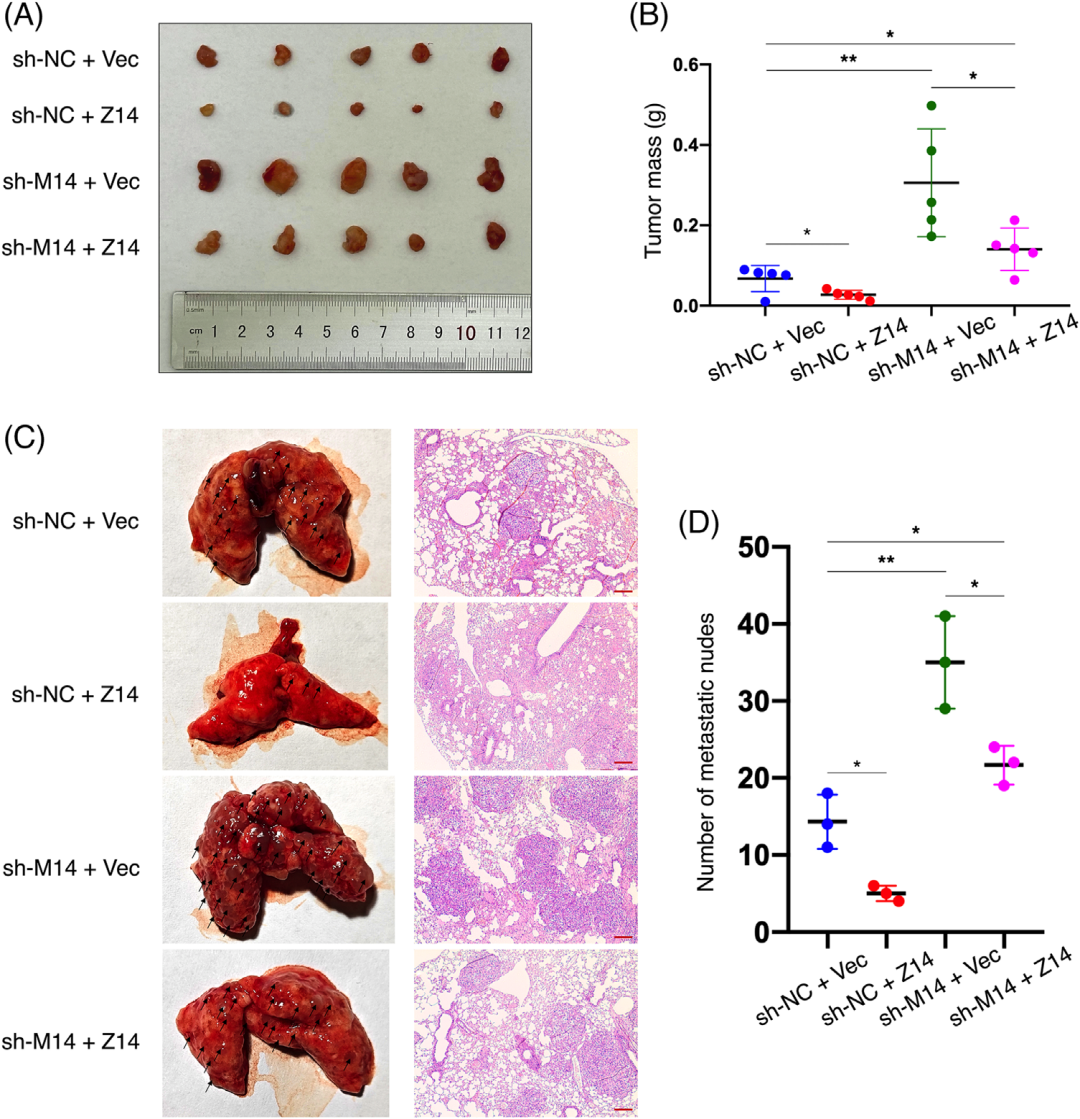
METTL14/ZFP14 axis inhibits in vivo proliferation and metastasis of ccRCC cells. (A) Subcutaneous tumours excised from BALB/c‐nude mice injected with ACHN cells of the indicated treatments. *n* = 5. (B) Weights of the subcutaneous tumours; *n* = 5. (C) Gross specimen and HE‐staining images of lungs with metastatic tumours excised from BALB/c‐nude mice injected with ACHN cells of the indicated treatments. *n* = 3. Bar scale = 50 µm. (D) Quantifications of the lung metastatic sites; *n* = 3. Data are presented as means ± SD. Student's *t*‐test was used for analyses. *p < .05, **p < .01.

Animal experiments were conducted using ACHN cells treated as described for the in vitro assays. Initially, in mice that underwent subcutaneous injection of these cells, over‐expression of ZFP14 resulted in lower tumour volumes and weights, while deficiency of METTL14 produced an opposite effect which was reversed by augmenting ZFP14 (Figure 5A,B). ZFP14 levels were assessed in the transplanted tumours by western blot assay (Figure S6). Additionally, in a tail vein‐lung metastasis model, enrichment of ZFP14 led to fewer lung metastatic sites and mitigated the metastasis promotion caused by silenced METTL14 (Figure 5C,D).

In summary, these findings identify ZFP14 as not only a tumour‐suppressor in ccRCC but also an important participant for METTL14 to inhibit ccRCC progression.

### 3.6 ZFP14 negatively regulates MMP1/3 expressions in ccRCC

**FIGURE 6:**
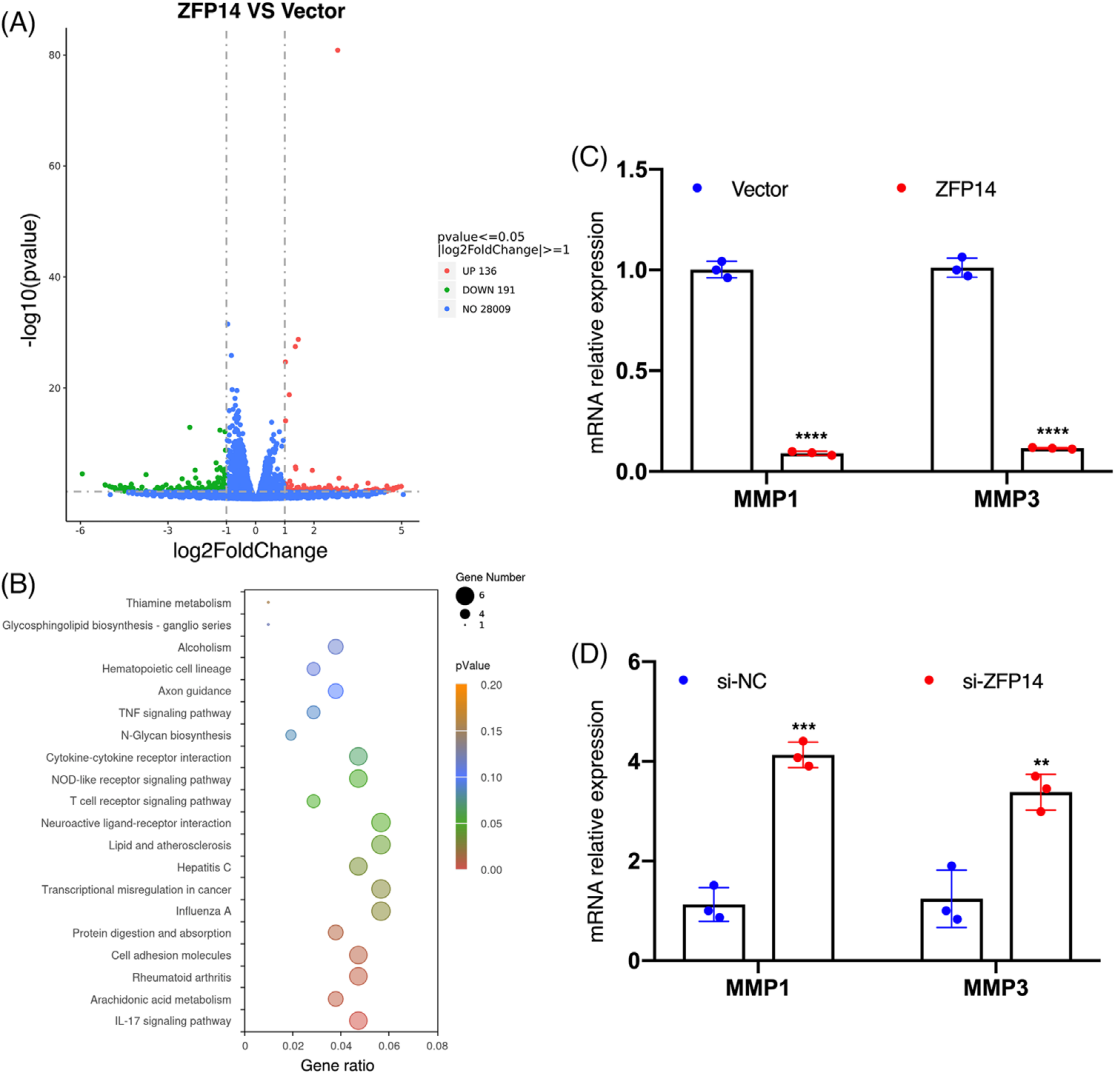
ZFP14 inhibits MMP1/3 expressions in ccRCC. (A) Volcano plots of DEGs detected by the mRNA‐seq. (B) The result of KEGG analyses for the DEGs detected by the mRNA‐seq. (C and D) Alterations of MMP1/3 mRNA levels in the indicated cells after ZFP14 over‐expression (C) and ZFP14 knockdown (D) detected by qRT‐PCR assay. Data are presented as means ± SD from three independent experiments. Student's *t*‐test was used for analyses. |log2FC| > 1 and *p* < .05 as the cutoff values for DEG filtration. **p < .01, ***p < .001 and ****p < .0001.

To further investigate the molecular behaviours of ZFP14 in ccRCC, we initially examined its endogenous expression in Caki‐1 versus ACHN cells. It was observed that ZFP14 levels were comparatively lower in Caki‐1 cells (Figure S7A). Upon over‐expressing ZFP14 in Caki‐1 cells (Figure S7B), an mRNA sequencing analysis was conducted. This analysis identified 327 DEGs, comprising 136 up‐regulated and 191 down‐regulated genes (Figure 6A). The Kyoto Encyclopedia of Genes and Genomes (KEGG) pathway analysis of these DEGs is shown in Figure 6B. Among these DEGs, matrix metalloproteinase 3 (MMP3) was notably under‐expressed to the greatest extent following ZFP14 enrichment; additionally, MMP1, another member of the MMP family, exhibited the maximum reduction in abundance among the DEGs with adjusted *p* values lower than .05 (Table S2). The MMP family is widely recognised as a critical regulator of cell adhesion, particularly in tumourigenesis and cancer progression.^18^ MMP1, universally over‐expressed across all malignancies,^19^ is identified as a potent oncogene and a key biomarker in ccRCC,^20^, ^21^ while MMP3 serves as an important indicator of malignant progression.^22^ To validate the sequencing results, qRT‐PCR assays were performed on these two DEGs. Results confirmed that ZFP14 over‐expression in Caki‐1 cells reduced mRNA levels of MMP1/3, whereas knocking down ZFP14 in ACHN cells produced the opposite effect (Figures 6C,D and S7C). Overall, ZFP14 exerts an inhibitory effect on MMP1/3 expressions in ccRCC and potentially influences a variety of DEGs as well as biological processes.

### 3.7 ZFP14 inhibits STAT3 expression in ccRCC via a ubiquitination‐dependent manner

**FIGURE 7:**
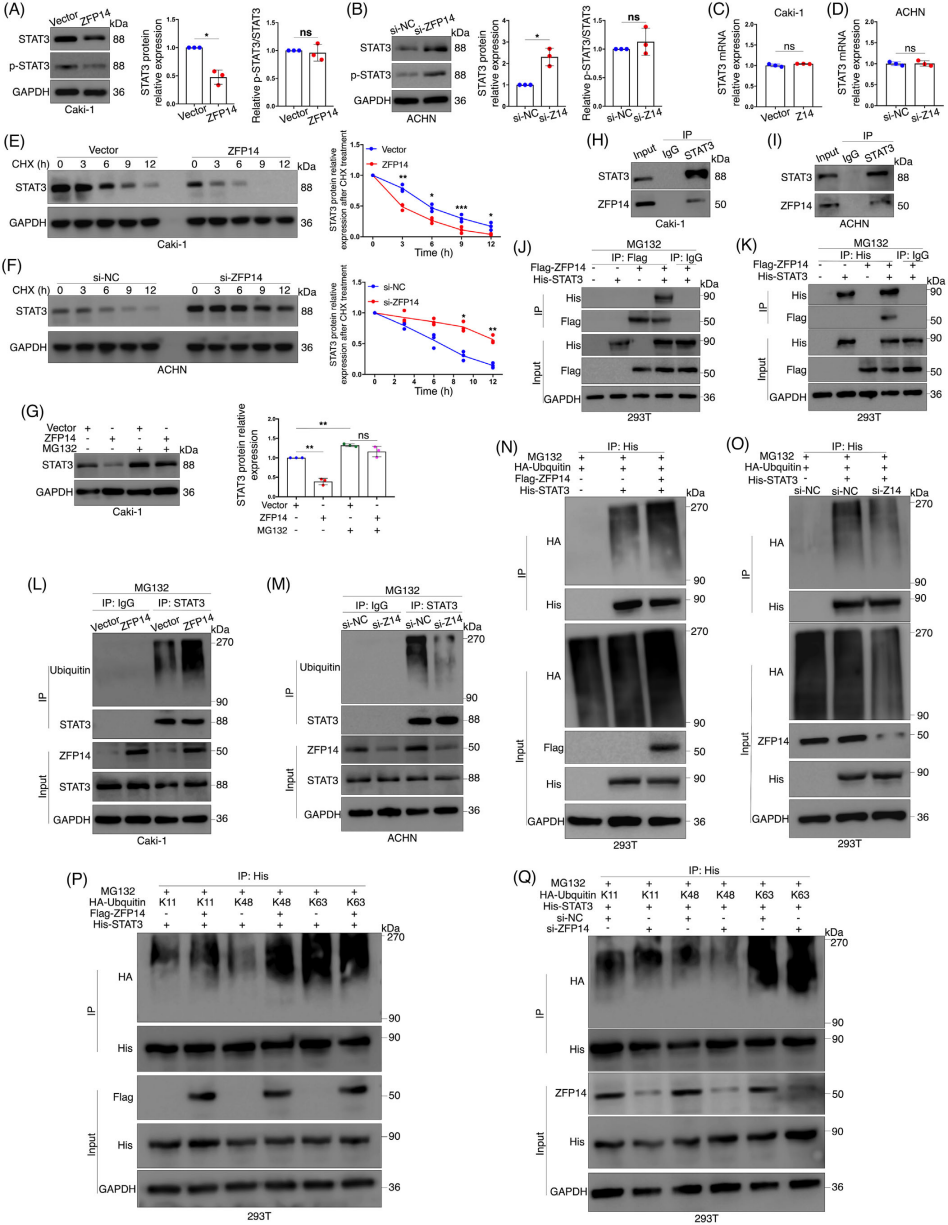
ZFP14 destabilises STAT3 via the ubiquitin–proteasome pathway. (A and B) Alterations of STAT3 and phosphorylated‐STAT3 protein levels in ccRCC cells after ZFP14 over‐expression (A) and ZFP14 knockdown (B) detected by western blot assay. (C and D) Alteration of STAT3 mRNA level in ccRCC cells after ZFP14 over‐expression (C) and ZFP14 knockdown (D) detected by qRT‐PCR. (E and F) Alterations of STAT3 protein stabilities after ZFP14 over‐expression (E) and knockdown (F) detected by protein stability assay. (G) STAT3 protein levels in Caki‐1 cells of the indicated disposals detected by western blot assay. (H and I) Endogenous interaction between ZFP14 and STAT3 in Caki‐1 (H) and ACHN (I) cells detected by Co‐IP assay. (J and K) Exogenous interaction between Flag‐ZFP14 and His‐STAT3 in HEK293T cells detected by Co‐IP assay respectively using Flag‐tag (J) and His‐tag (K) antibodies for immunoprecipitations. (L and M) Alterations of ubiquitination levels of endogenous STAT3 in ccRCC cells after ZFP14 over‐expression (L) and ZFP14 knockdown (M) detected by ubiquitination assay. (N and O) Alterations of His‐STAT3 exogenous ubiquitination levels in HEK293T cells after Flag‐ZFP14 transfection (N) and ZFP14 knockdown (O) detected by ubiquitination assay. (P and Q) Alterations of His‐STAT3 exogenous ubiquitination of the indicated linkages in HEK293T cells after Flag‐ZFP14 transfection (P) and ZFP14 knockdown (Q) detected by ubiquitination assay. Data are presented as means ± SD from three independent experiments. Student's *t*‐test was used for analyses. *p < .05, **p < .01 and ***p < .001. ns, non‐significant.

Although not widely reported, ZFP14 has been recently identified as a key component of an E3 ubiquitin ligase complex, promoting the ubiquitination and decay of downstream protein.^14^ Therefore, it was speculated that ZFP14 might exert similar effects on specific proteins, subsequently regulating the transcript levels of the DEGs in ccRCC cells. Recently, some literature has identified impaired ubiquitination of STAT3 as a key factor in the tumourigenesis and progression of malignancies,^23^, ^24^ but it has not been illustrated in ccRCC. Additionally, STAT3 is widely recognised as a regulator of MMP1/3,^25^, ^26^, ^27^ leading to our interest in whether it could be a target of ZFP14‐controlled ubiquitination. Western blot analyses revealed that over‐expression and knockdown of ZFP14 respectively decreased and increased STAT3 protein levels in ccRCC cells, without affecting its phosphorylation status (Figure 7A,B). However, changes in ZFP14 abundance did not affect STAT3 mRNA levels, suggesting a posttranscriptional regulation (Figure 7C,D). Subsequently, results of protein stability assay demonstrated a significant acceleration of STAT3 protein degradation following ZFP14 over‐expression in Caki‐1 cells, while silencing ZFP14 increased STAT3 stability (Figure 7E,F). Furthermore, treatment of Caki‐1 cells with the proteasome inhibitor MG132 led to an increase in STAT3 levels; MG132 treatment also could negate the inhibitory effect of ZFP14 over‐expression on STAT3 protein expression (Figure 7G). These findings indicated that ZFP14 promoted STAT3 decay via the proteasome pathway.

To validate whether ZFP14 regulated STAT3 expression through ubiquitination, a Co‐IP assay was first conducted, confirming the interaction between these two proteins in both Caki‐1 and ACHN cells (Figure 7H,I). Additionally, the interaction between exogenous Flag‐ZFP14 and His‐STAT3 was verified by Co‐IP assay following co‐transfection in HEK293T cells (Figure 7J,K). Subsequent ubiquitination experiments demonstrated that increased ZFP14 expression enhanced the ubiquitination of STAT3 in Caki‐1 cells, whereas ZFP14 knockdown in ACHN cells had the opposite effect (Figure 7L,M). For His‐STAT3 in HEK293T cells, ZFP14 positively regulated its exogenous ubiquitination level (Figure 7N,O). Ubiquitin residues link in various ways, with polyubiquitination types including K11, K48 and K63 being the most abundant and studied.^28^ HA‐tagged ubiquitin plasmids of these types were transfected into HEK293T cells along with His‐STAT3, after which ZFP14 was either over‐expressed or silenced and ubiquitination assays were performed. The results showed that ZFP14 over‐expression increased and its knockdown decreased HA‐K48 ubiquitin on His‐STAT3, without affecting HA‐K11 or HA‐K63 levels (Figure 7P,Q).

Overall, ZFP14 promotes K48‐linked ubiquitination of STAT3, accelerating its decay via the ubiquitin–proteasome system in ccRCC.

### 3.8 ZFP14 inhibits ccRCC progression induced by STAT3

**FIGURE 8:**
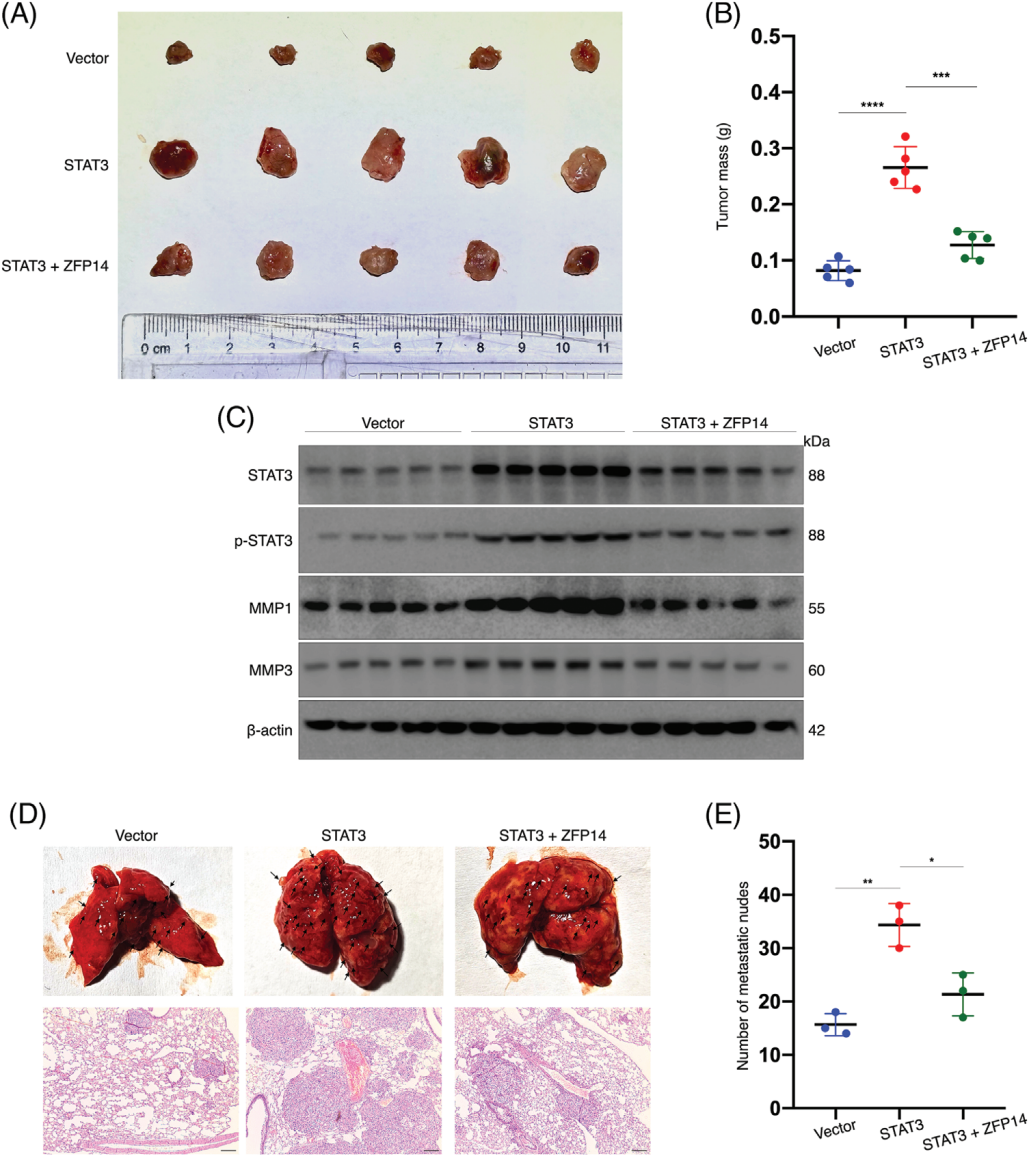
ZFP14 inhibits in vivo proliferation and metastasis of ccRCC cells by under‐expressing STAT3. (A) Subcutaneous tumours excised from BALB/c‐nude mice injected with ACHN cells of the indicated treatments; *n* = 5. (B) Weights of the obtained subcutaneous tumours; *n* = 5. (C) Protein levels of STAT3, phosphorylated STAT3 and MMP1/3 in the obtained subcutaneous tumours; *n* = 5. (D) Gross specimen and HE‐staining images of lungs with metastatic tumours excised from BALB/c‐nude mice injected with ACHN cells of the indicated treatments; *n* = 3. Bar scale = 50 µm. (D) Quantifications of the lung metastatic sites; *n* = 3. Data are presented as means ± SD. Student's *t*‐test was used for analyses. *p < .05, **p < .01, ***p < .001 and ****p < .0001.

To explore the impact of ZFP14‐mediated regulation of STAT3 on ccRCC biological behaviours, in vivo rescue assays were performed. ACHN cells were transfected as shown in Figure S8, where ZFP14 over‐expression could rescue the effect of elevated STAT3. In the subcutaneous xenograft model, STAT3 over‐expression increased tumour sizes and weights as well as MMP1/3 expressions, but these effects were reversed by ZFP14 over‐expression; besides, the change of phosphorylated STAT3 level in the tumours was basically accordant to that of the total STAT3 expression (Figure 8A–C). Additionally, ZFP14 mitigated the STAT3‐enhanced tumour metastasis (Figure 8D,E). Thus, it is concluded that ZFP14 impedes ccRCC progression by inhibiting STAT3 expression.

## 4 DISCUSSION

ccRCC, a prevalent and life‐threatening urologic malignancy, remains a significant challenge for urologists and oncologists. It is characterised by a lack of distinctive symptoms in its early stages and resistance to chemotherapy and radiotherapy. Furthermore, the efficacy of other systemic treatments for advanced ccRCC, including immunotherapy and targeted therapies, remains inconsistent and generally inadequate,^29^ highlighting the urgency of advancing our molecular understanding and identifying novel therapeutic targets. As the predominant RNA modification, m6A has been recognised for its crucial role in oncogenesis.^11^, ^30^ Although predominantly oncogenic, m6A was identified as a tumour suppressor in ccRCC in one of our previous studies.^13^ Consistent with other reports, we found that N6‐methyltransferase METTL14, which is notably under‐expressed in ccRCC, is a crucial m6A component influencing ccRCC progression.^12^, ^13^, ^17^ However, the intricate mechanisms by which METTL14‐mediated m6A functions in ccRCC remain to be elucidated, with further exploration needed for its significant downstream targets.

In the present study, ZFP14 was selected based on its significant increase in m6A abundance after METTL14 over‐expression, as shown by our MeRIP‐seq results. Although rarely reported, ZFP14 has been shown to stimulate the in vitro proliferation and migration of colorectal and breast cancer cells,^14^ but its role in ccRCC remains unexplored. Our bioinformatic analyses indicated a potential tumour‐suppressing role for ZFP14 in ccRCC, suggested by the positive correlation between its low expression, tumour progression and poor patient prognosis. However, the under‐expression of ZFP14 in tumour tissues was not statistically significant, likely due to comparisons with non‐paired normal tissues in the TCGA cohort. Nevertheless, by conducting qRT‐PCR and western blot assays using paired samples, we confirmed the significant reduction in both mRNA and protein levels of ZFP14 in ccRCC. Importantly, ZFP14 levels were negatively associated with ccRCC pathological grades and positively correlated with METTL14 and total m6A abundance. These results suggested not only the probable effect of ZFP14 under‐expression on ccRCC carcinogenesis and advancement but also a potential regulatory relationship between ZFP14 and METTL14‐mediated m6A. Further, we observed that METTL14 positively regulated ZFP14 protein and mRNA levels in ccRCC cells, confirming this writer as an upstream of ZFP14. m6A affects various biological features of its target mRNAs, notably stability, which predominates mRNA abundance.^31^ Additionally, two potential m6A modification sites on ZFP14 were identified within its 3′UTR, a region primarily associated with mRNA decay.^32^, ^33^ Consistent with our predictions, ZFP14 mRNA was stabilised by METTL14 in ccRCC cells. It further supported the possibility of METTL14 regulating ZFP14 expression via the m6A manner. Mechanistically, we confirmed that METTL14 directly interacted with both identified m6A sites on ZFP14 mRNA, increasing their m6A abundance. The fate of m6A‐modified RNA is dictated by various readers. For instance, YTHDF1, 3 and YTHDC2 enhance target RNA translation; YTHDF2 and 3 target mRNA for degradation; YTHDC1 and HNRNPC mediate splicing, while HNRNPA2B1 processes microRNA.^34^ The IGF2BPs are known to reinforce the stability of target transcripts, which led us to further examine their role.^34^ Through systemic investigation, the m6A reader IGF2BP2 was identified as a critical component in METTL14‐mediated m6A regulation of ZFP14 mRNA stability and expression. These findings not only validate our MeRIP‐seq results and expand our understanding of METTL14 and m6A mechanisms but also clarify the abnormal expression of ZFP14 in ccRCC. More importantly, the METTL14/ZFP14 axis was shown to suppress several malignant features of ccRCC cells, including proliferation, migratory ability, invasiveness and metastasis, offering new potential therapeutic strategies for this life‐threatening malignancy.

According to previous studies, although METTL14 significantly influences ccRCC biological behaviours, an increasing number of researchers have noted the involvement of other m6A regulators, each with distinct mechanisms.^35^, ^36^, ^37^ Additionally, in other diseases, it has been observed that multiple m6A factors can target a single downstream gene, either synergistically or antagonistically affecting its biochemical properties.^38^, ^39^ Furthermore, the 3′UTR of mRNA, a region rich in m6A, is known to play various roles beyond controlling mRNA stability.^40^, ^41^ Thus, the m6A modification of ZFP14, potentially involving more regulators and complex mechanisms, warrants further investigation. Also, considering ZFP14's role as a downstream target of TP53,^14^ it is plausible that multiple factors contribute to ZFP14's aberrant expression in ccRCC, not just m6A dysregulation.

In this study, ZFP14 was identified for the first time not only as a tumour suppressor in ccRCC but also as a crucial target of METTL14‐mediated m6A, with IGF2BP2 participating. This research provides deeper insights into the significant epitranscriptomic modification in ccRCC and holds practical and clinical relevance. Given m6A's oncogenic roles in many cancers,^11^ the development of inhibitors targeting m6A writers, including METTL14, has garnered substantial interest for cancer therapy, leading to remarkable progress.^42^, ^43^, ^44^ However, our findings reiterate the complex and unique role of m6A in ccRCC, highlighting the necessity of carefully considering safety before the clinical application of these inhibitors.

By using mRNA‐seq, we identified numbers of DEGs in ccRCC cells potentially regulated by ZFP14, among which the oncogenes MMP1 and MMP3 were confirmed to be inhibited at the transcript level. This further indicated ZFP14’ as a tumour suppressor in ccRCC. Given ZFP14's role in the MDM2 E3 ubiquitin ligase complex,^14^ we hypothesised that it targets certain proteins, thereby altering the mRNA expressions of these DEGs. Our experiments showed that STAT3, a common regulator of MMP1/3, was directly destabilised by ZFP14 in a ubiquitination‐dependent manner. Additionally, other DEGs such as ICAM5, ETV4, DUSP4 and SAA1, previously reported to be regulated by STAT3,^45^, ^46^, ^47^, ^48^ support the role of STAT3 as an intermediary through which ZFP14 affects DEG expressions. Recent studies have linked cancer progression to the reduced ubiquitination and subsequent over‐expression of STAT3,^23^, ^24^, ^49^ identifying it as a potential therapeutic target for some malignancies.^50^, ^51^, ^52^, ^53^ In 2019, it was reported that STAT3 destabilisation, following SIRT3‐mediated deacetylation, suppressed ccRCC tumourigenesis.^54^ However, it remains unverified whether the disorder of STAT3 stability results from ubiquitination dysregulation in this malignancy. Our research provides new insights into the abnormal expression of STAT3 and unveils, for the first time, the molecular mechanisms of ZFP14 in ccRCC, expanding our understanding of this rarely‐reported protein and ubiquitination processes. Currently, ZFP14 is not specifically identified with ubiquitinase activity and is likely to regulate STAT3 ubiquitination through dependency on an E3 ubiquitin ligase or a deubiquitinase, similar to how it affects TP53 stabiliy.^14^ Future studies are expected to identify these potential collaborators and further important target proteins of ZFP14. Additionally, it is worth exploring whether ZFP14‐mediated ubiquitination affects other biological characteristics of STAT3, such as its intracellular location. Finally, we observed that ZFP14 inhibited ccRCC growth and metastasis as well as reducing MMP1/3 transcript levels by suppressing STAT3, supporting our hypothesis and highlighting the potential therapeutic value of ZFP14.

In this research, we identified ZFP14 as a tumour suppressor in ccRCC, characterised by its reduced expression, suggesting its potential as a new diagnostic biomarker. Mechanistically, the under‐expression of ZFP14 was attributed to the impaired m6A modification involving a novel collaboration between METTL14 and IGF2BP2 specific to ccRCC. Additionally, ZFP14 was shown to destabilise STAT3 via the ubiquitin–proteasome pathway. To date, most studies on the correlation between m6A and STAT3 have focused on the activation or inactivation of STAT3 and its pathways.^55^, ^56^, ^57^, ^58^, ^59^ While interest in STAT3 ubiquitination has been growing, it has not been systematically investigated in ccRCC. Thus, our findings introduce some novelty and advancement. Hundreds of DEGs identified from the sequencing indicate ZFP14's complex characteristics and potential for further research. Most importantly, the significant biological impacts of the METTL14/ZFP14 axis and ZFP14/STAT3 axis provide valuable insights for ccRCC therapy, which was the original intention of this study. In recent years, the technique of adeno‐associated virus (AAV) has been successfully developed for gene delivery and used in therapeutic trials for various diseases,^60^, ^61^, ^62^ and could potentially be used to restore METTL14 and ZFP14 in ccRCC in the future. However, additional studies are necessary to confirm the molecular mechanisms and functions, such as the use of a transgenic animal model and inclusion of more clinical sample from multiple centres for validations. Subsequent translational research will be crucial for clinical application.

**FIGURE 9:**
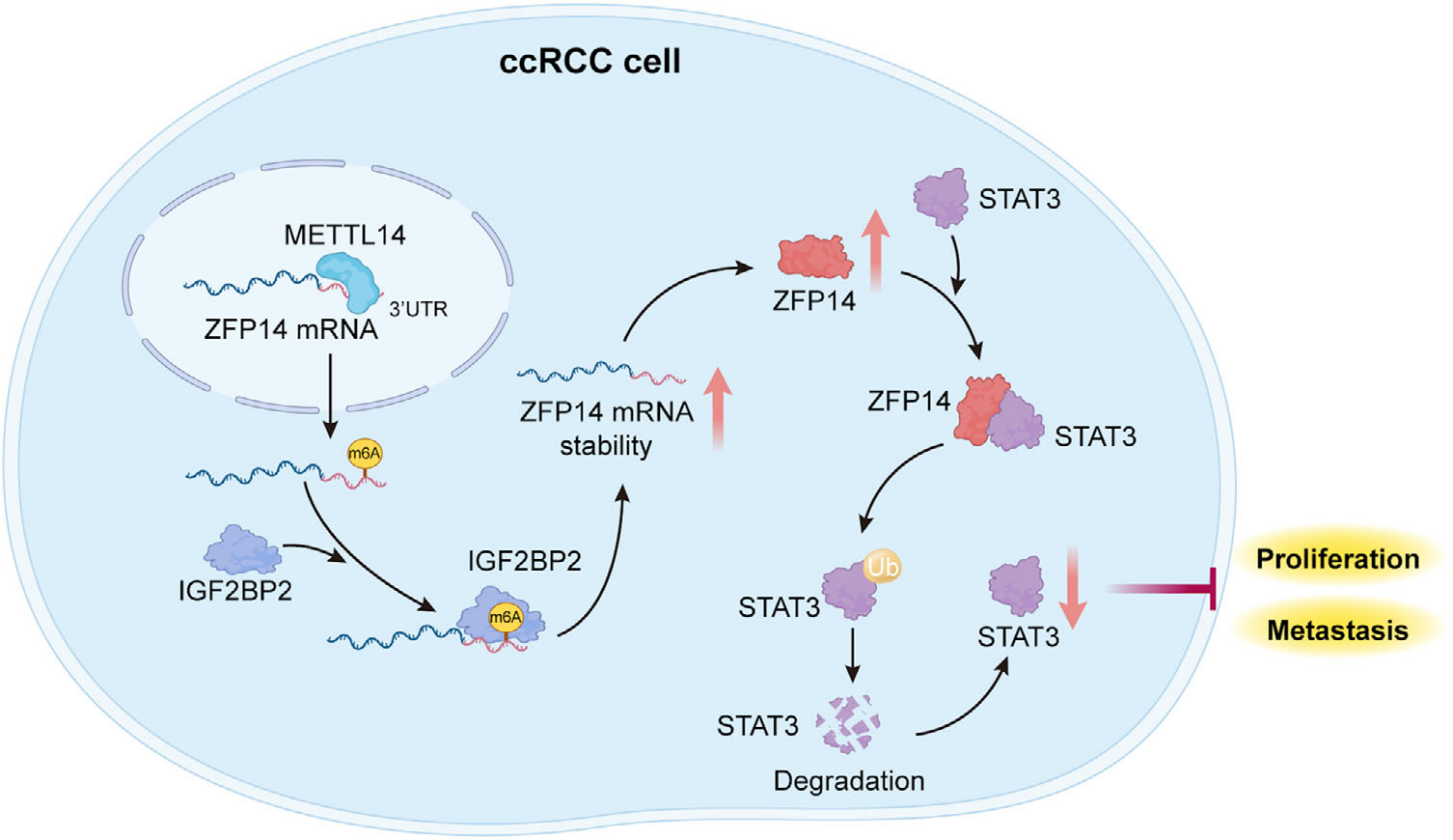
Proposed working model of the demonstrated mechanisms in this study.

In summary, with the assistance of IGF2BP2 as the reader, METTL14‐mediated m6A was shown to over‐express ZFP14, which could destabilise STAT3 protein via the ubiquitin–proteasome pathway, inhibiting the progression of ccRCC (Figure 9). These findings will hopefully contribute to further investigations into the biological mechanisms of ccRCC and the development of promising diagnostic and therapeutic strategies for this malignancy.

## AUTHOR CONTRIBUTIONS

Z. L. conceived and designed the study and performed most of the experiments. T. S. assisted in the bioinformatic and statistical analyses. C. P. conducted the animal experiments. T. S., Z. Z., C. K. and T. Z. contributed to the regents and materials. Z. L. wrote the paper. C. K. and T. Z. supervised the research.

## CONFLICT OF INTEREST STATEMENT

The authors declare no conflicts of interest.

## ETHICS STATEMENT

The use of clinical samples for the present study was approved by The Ethics Committee of the First Hospital of China Medical University (NO. 2022‐48‐2) and all the participants had hand‐written informed consent before enrolment. The Animal Ethics and Welfare Committee of China Medical University had provided approval for all the animal experiments performed (NO. CMUKT2023265).

## Supporting information



Supporting information

Supporting information

Supporting information

## Data Availability

The MeRIP‐seq and mRNA‐seq data involved in this study have been respectively deposited in the NCBI Sequence Read Archive (SRA) with BioProject ID PRJNA1062284 and PRJNA1059280. All the other data used and/or analysed for the current study are available from the corresponding author on reasonable request.
