# Supplementary material for: METTL14‐mediated m6A modification of ZFP14 inhibits clear cell renal cell carcinoma progression via promoting STAT3 ubiquitination
Source: Clin Transl Med. 2025 Feb 12;15(2):e70232. doi: 10.1002/ctm2.70232 (PMC11815563; doi:10.1002/ctm2.70232)
Supplement: Supplementary file 3 — Supporting information [file CTM2-15-e70232-s002.docx]

Supplementary materials

METTL14-mediated m6A modification of ZFP14 inhibits clear cell renal cell carcinoma progression via promoting STAT3 ubiquitination

Running title: ZFP14 inhibits ccRCC progression

Zhuonan Liu^1^, Tianshui Sun^2^, Zhe Zhang^1^, Chiyuan Piao^1^, Chuize Kong^1^* and Xiaotong Zhang^1^*

^1^ Department of Urology, First Hospital of China Medical University; No. 155 Nanjing North Street, Heping District, Shenyang City, Liaoning Province, 110004, P.R. China.

^2^ Department of Obstetrics and Gynecology, Shengjing Hospital of China Medical University, Shenyang, Liaoning 110004, P.R. China.

ZL: liuzhn_cmu@163.com; TS: tssun1995@163.com; ZZ: [63099390@qq.com](mailto:63099390@qq.com); CP: [cypiao@cmu.edu.cn](mailto:cypiao@cmu.edu.cn).

*Correspondence: [kongchuize_cmu@sina.cn](mailto:kongchuize_cmu@sina.cn); [pethoid@126.com](mailto:pethoid@126.com). Department of Urology, First Hospital of China Medical University; No. 155 Nanjing North Street, Heping District, Shenyang City, Liaoning Province, 110004, P.R. China

**List of Supplementary Materials**

Figure S1-S8

Table S3-S7


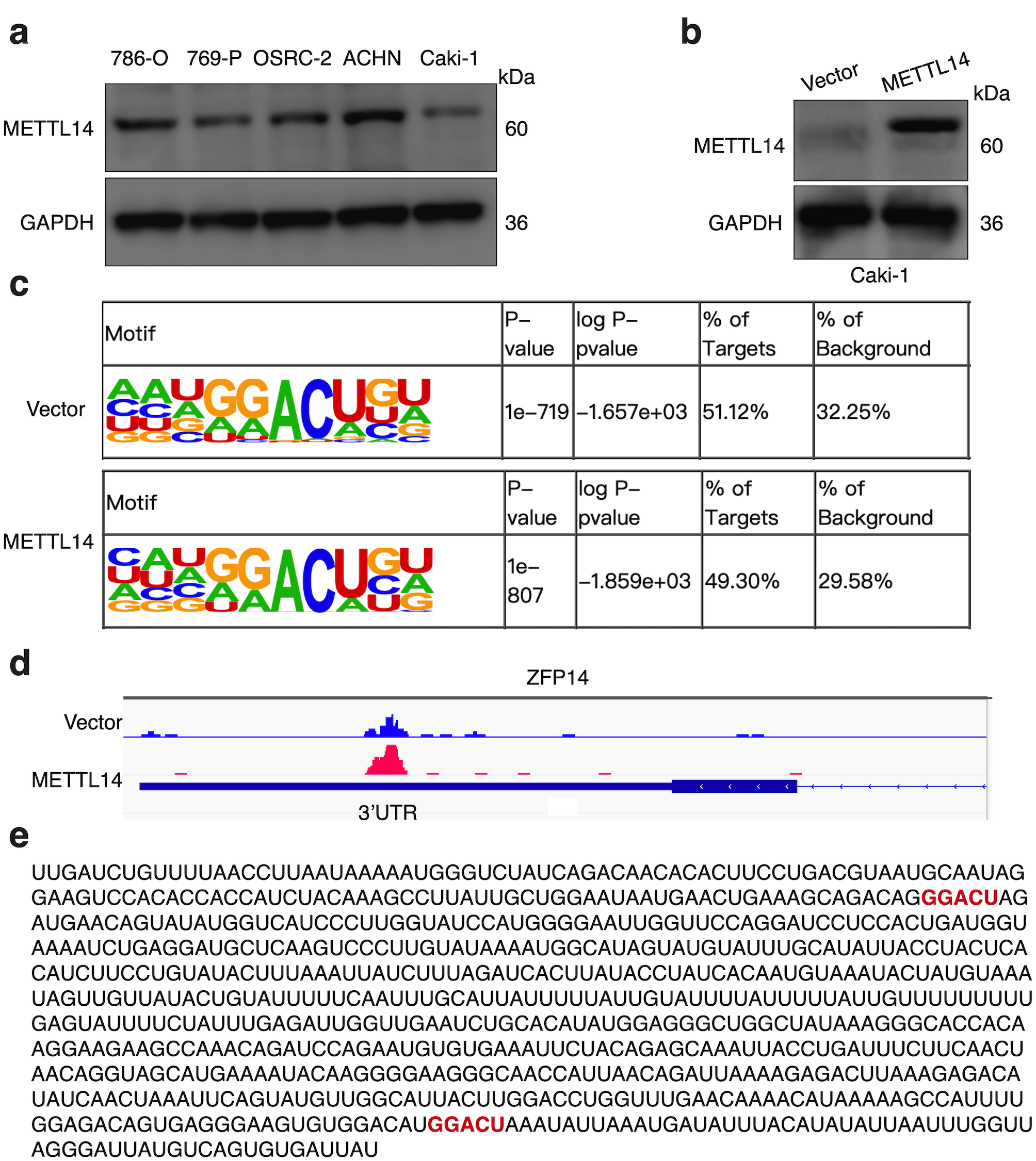


**Figure S1** ZFP14 is a newly identified target of METTL14-mediated m6A in ccRCC. **a** Protein levels of ZFP14 in the indicated ccRCC cell lines detected by western blot assay. **b** ZFP14 protein levels in Caki-1 cells of the indicated disposals detected by western blot assay. **c** Predominant consensus motif of m6A detected in Caki-1 cells respectively overexpressing empty vector and METTL14. **d** m6A peak enrichments in ZFP14 3’UTR in the indicated cells visualized by the Integrative Genomics Viewer. m6A level comparison (METTL14 versus Vector): lg FDR = -1.92 and lg *p* = -3.27. **e** m6A motifs detected in ZFP14 3’UTR sequence. Student’s t test was used for analyses. FDR < 0.05 and *p* < 0.05 are considered statistically significant.


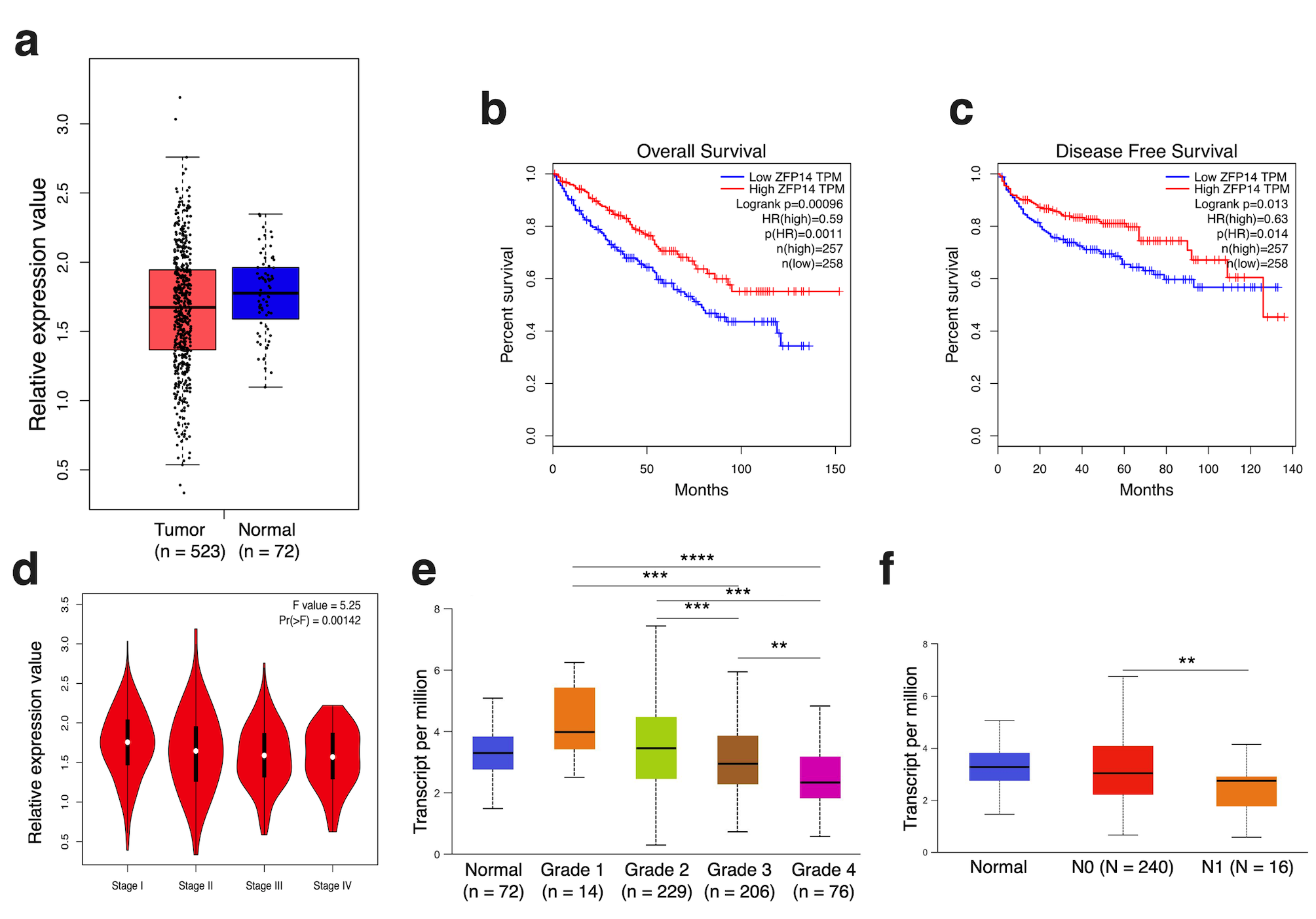


**Figure S2** Under-expression of ZFP14 is associated with ccRCC tumorigenesis and progression. **a** ZFP14 mRNA levels in ccRCC tissues (n = 523) and normal kidney tissues (n = 72) in TCGA database. The result was downloaded from GEPIA (http://gepia.cancer-pku.cn/). Student’s t test was used**. b, c** Kaplan–Meier analysis of the TCGA ccRCC patients’ overall survival (**b**) and disease-free survival (**c**) based on ZFP14 mRNA levels. The result was downloaded from GEPIA. log-rank test was used. **d** ZFP14 mRNA levels in ccRCC of different stages. The result was downloaded from GEPIA. ANOVA test was used. **e, f** ZFP14 mRNA levels in ccRCC of different pathological grades (**e**) and metastasis status (**f**) in TCGA database. The result was downloaded from UALCAN (<http://ualcan.path.uab.edu/>). Student’s t test was used to evaluate significance between two groups. ANOVA test assessed the difference among multiple groups. Survival rates were analyzed using the log-rank test *p* < 0.05 is considered significant statistically. **p* < 0.05, ***p* < 0.01, ****p* < 0.001 and *****p* < 0.0001.


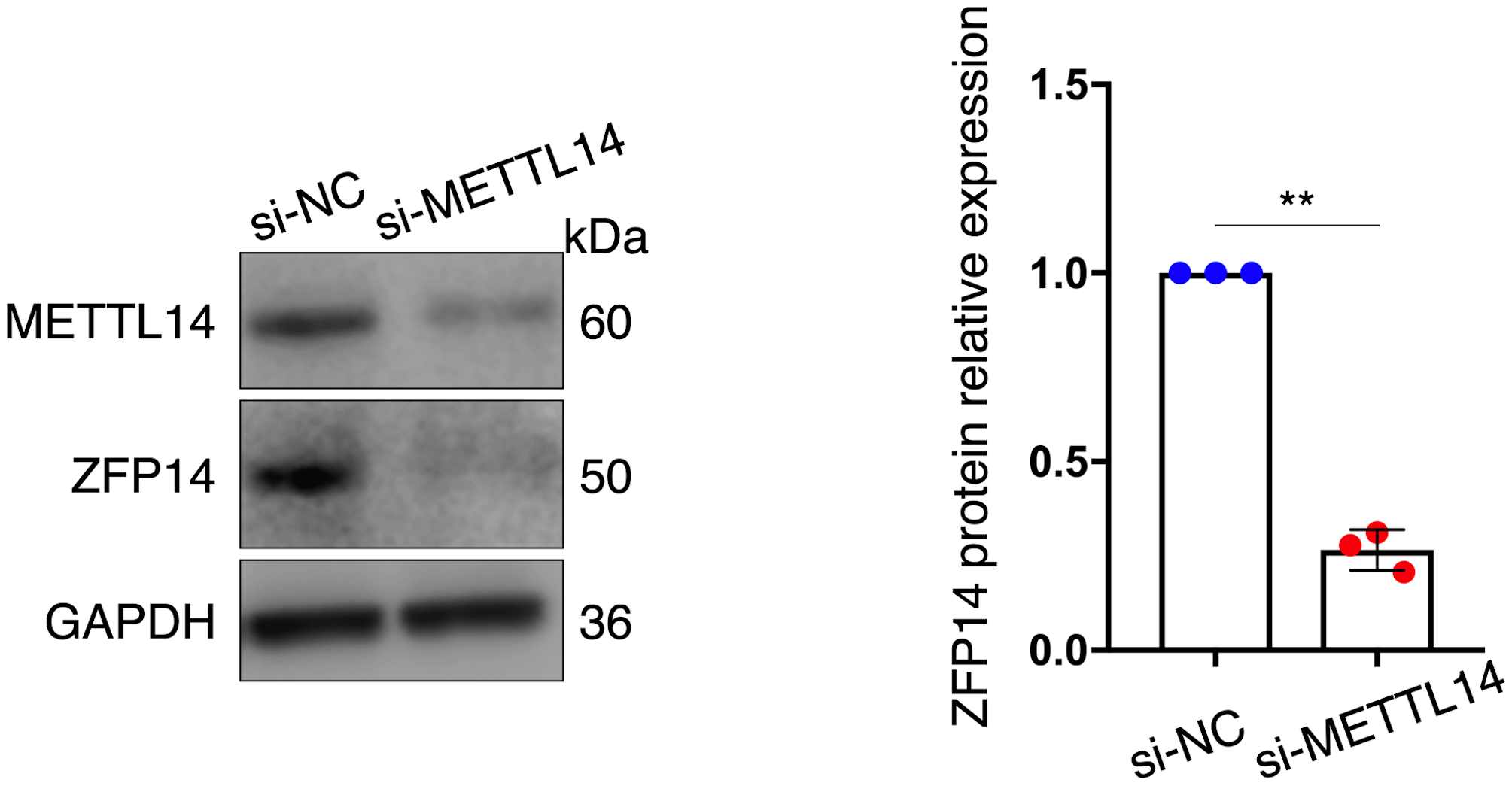


**Figure S3** Alterations of METTL14 and ZFP14 protein levels after METTL14 knockdown in the ACHN cells detected by western blot assay. Data are presented as means ± SD based on triple independent experiments. Student’s t test was used for analyses. ***p* < 0.01.


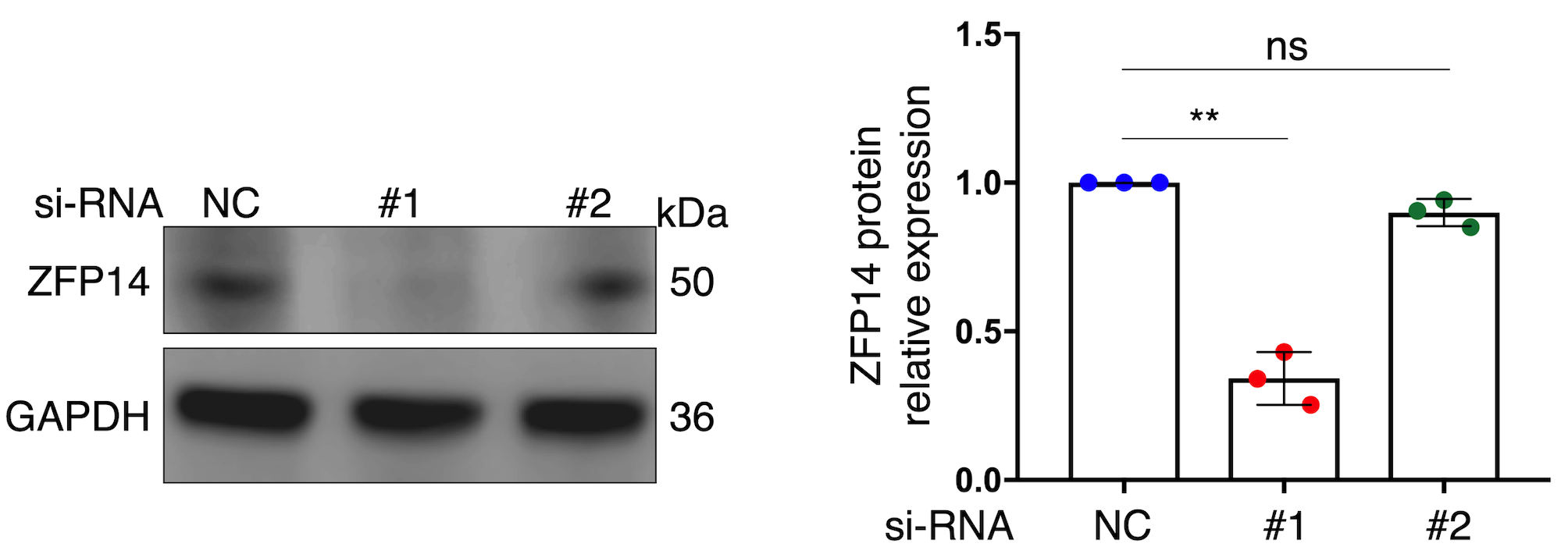


**Figure S4** ZFP14 protein levels in Caki-1 cells of the indicated treatments detected by western blot assay. Data are presented as means ± SD based on triple independent experiments. Student’s t test was used for analyses. ***p* < 0.01. ns, non-significant.


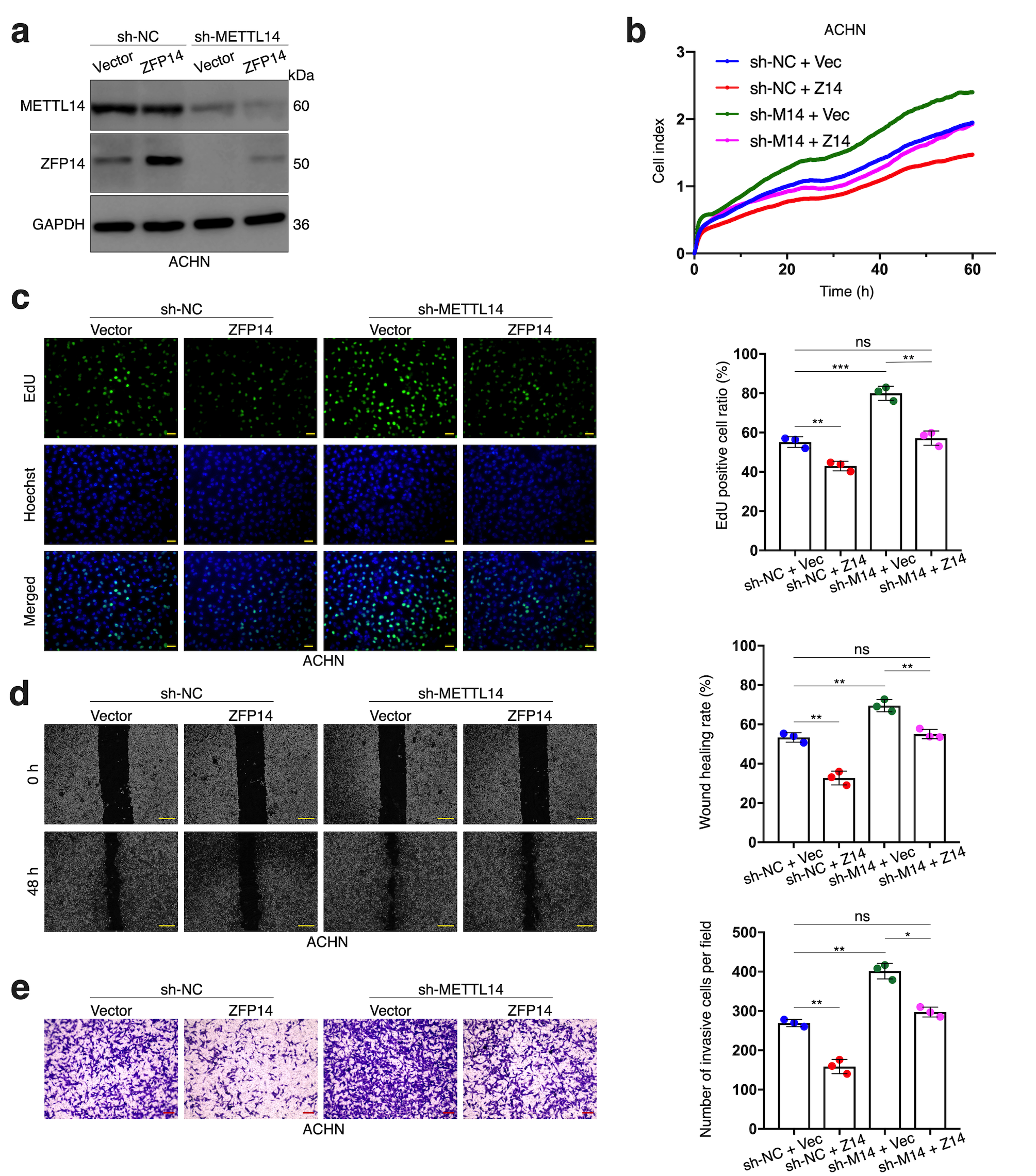


**Figure S5** METTL14/ZFP14 axis inhibits ccRCC cell *in vitro* proliferation, migration, and invasion. **a** METTL14 and ZFP14 protein levels in ACHN cells of the indicated disposals detected by western blot assay. **b** Proliferation curves of ACHN cells of the indicated disposals detected by RTCA assay. **c** Proliferation status of ACHN cells of the indicated disposals detected by EdU assay. Bar scale = 20 μm. **d** Migratory rates of ACHN cells of the indicated disposals detected by wound-healing assay. Bar scale = 50 μm. e Invasiveness of Caki-1 cells of the indicated disposals detected by cell invasion assay. Bar scale = 50 μm. Data are presented as means ± SD from three independent experiments. Student’s t test was used for analyses. **p* < 0.05, ***p* < 0.01 and ****p* < 0.001. ns, non-significant.


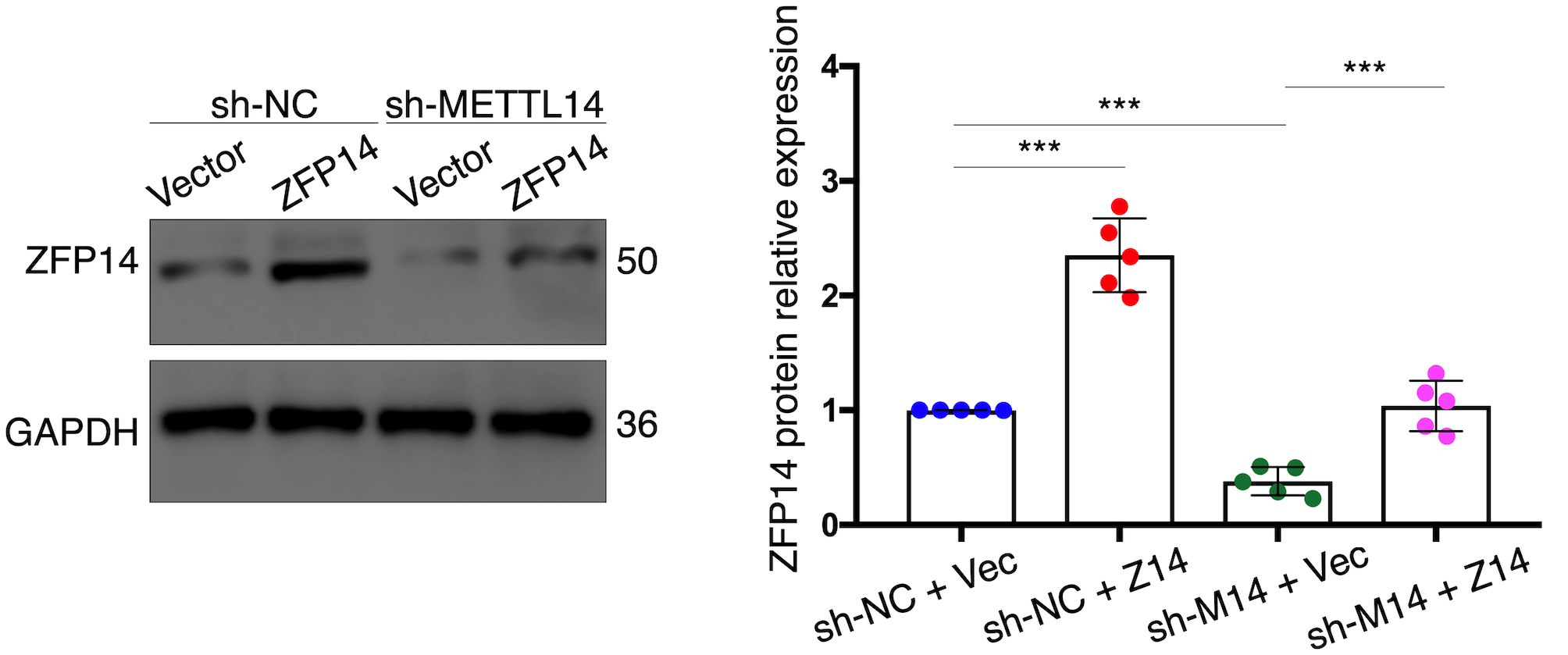


**Figure S6** ZFP14 protein levels in the subcutaneous transplanted tumors detected by western blot assay. n = 5. Data are presented as means ± SD. ****p* < 0.001.


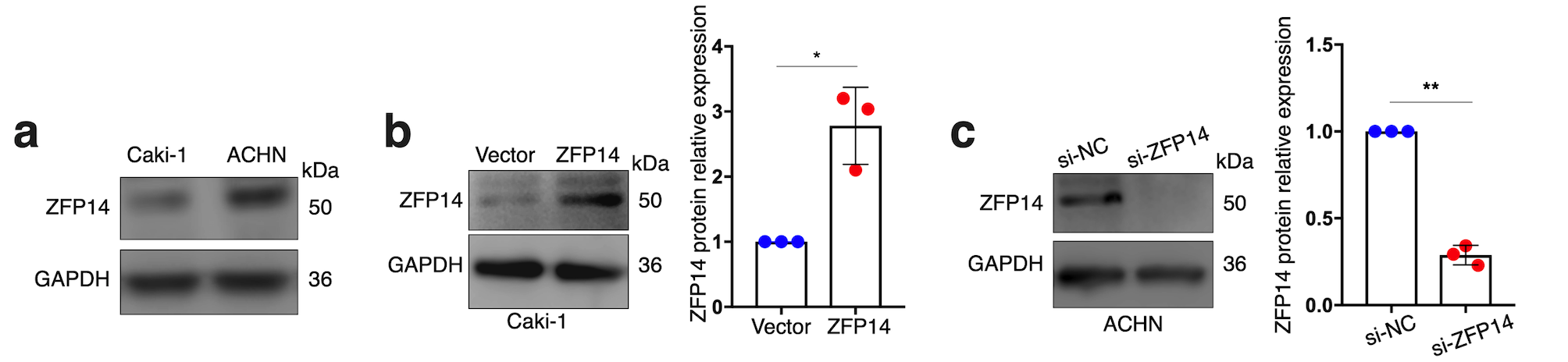


**Figure S7 a** ZFP14 protein levels in Caki-1 and ACHN cells detected by western blot assay. **b, c** Alterations of ZFP14 protein levels by ZFP14 overexpression (**b**) and knockdown (**c**) detected by western blot assay. Data are presented as means ± SD from three independent experiments. Student’s t test was used for analyses. **p* < 0.05, ***p* < 0.01.


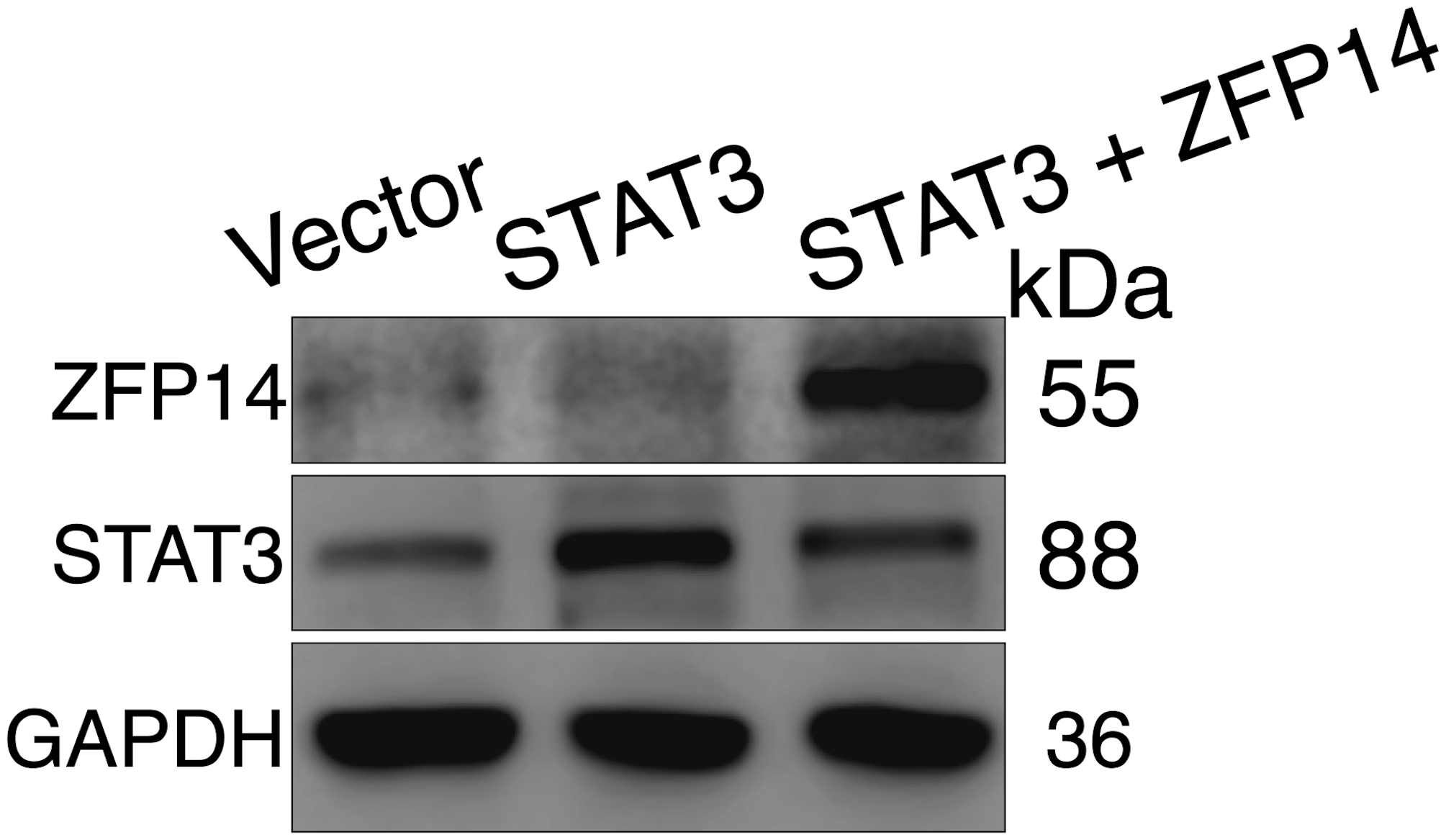


**Figure S8** ZFP14 and STAT3 protein levels in ACHN cells of the indicated treatments detected by western blot assay.

**Table S3** siRNA sequences

| siRNA | Sense sequences | Anti-sense sequences |
| --- | --- | --- |
| si-METTL14 | GGAUGAAGGAGAGACAGAU | CCUGGGAAGACUAAGACUU |
| si-IGF2BP1 | CCGGGAAAGUAGAAUUACAAGGAAA | UUUCCUUGUAAUUCUACUUUCCCGG |
| si-IGF2BP2 | CAGUUUGUUGGUGCCAUCAUCGGAA | UUCCGAUGAUGGCACCAACAAACUG |
| si-IGF2BP3 | CCCUCGGACCUAGAAAGUAUCUUCA | UGAAGAUACUUUCUAGGUCCGAGGG |
| si-ZFP14#1 | UUAGAACUGCAAAAACUGGUC | CCAGUUUUUGCAGUUCUAAAA |
| si-ZFP14#2 | AAUGAAGUUGCUGUAGUUCUC | GAACUACAGCAACUUCAUUUC |

**Table S4** shRNA sequences

| shRNA | Sequences |
| --- | --- |
| sh-METTL14 | GCATTGGTGCCGTGTTAAATATATTTAACACGGCACCAATGC |
| sh-IGF2BP2 | TGGAATTGCATGGGAAAATCATG |

**Table S5** Sequences of qRT-PCR primers

| Primers | Forward sequences | Reverse sequences |
| --- | --- | --- |
| ZFP14 | GGAAAGCCTTCGTATGTGGTC | TGATGAACAGTAAGTTGTTGGCA |
| ZFP14 3’UTR m6A site 1 | CAATAGGAAGTCCACACCACCA | AGTCCCTGTCTGCTTTCAGTT |
| ZFP14 3’UTR m6A site 2 | GAGGGAAGTGTGGACATGGAC | AGTTTCCAGCTCTCTTTGCCAAT |
| MMP1 | CTCTGGAGTAATGTCACACCTCT | TGTTGGTCCACCTTTCATCTTC |
| MMP3 | AGTCTTCCAATCCTACTGTTGCT | TCCCCGTCACCTCCAATCC |
| STAT3 | ATCACGCCTTCTACAGACTGC | CATCCTGGAGATTCTCTACCACT |
| β‐actin | CATGTACGTTGCTATCCAGGC | CTCCTTAATGTCACGCACGAT |
| GAPDH | ACAACTTTGGTATCGTGGAAGG | GCCATCACGCCACAGTTTC |

**Table S6** Information of primary antibodies

| Antibodies | Source | Identifier |
| --- | --- | --- |
| ZFP14 | NOVUS | Cat# NBP1-79388, RRID: AB_11004822 |
| METTL14 | Proteintech | Cat# 26158-1-AP, RRID: AB_2800447 |
| IGF2BP1 | Proteintech | Cat# 22803-1-AP, RRID: AB_ 2879173 |
| IGF2BP2 | Proteintech | Cat# 11601-1-AP, RRID: AB_2122672 |
| IGF2BP3 | Proteintech | Cat# 14642-1-AP RRID: AB_2122782 |
| STAT3 | Proteintech | Cat# 10253-2-AP, RRID: AB_2302876 |
| p-STAT3 | Abcam | Cat# ab32143, RRID: AB_2286742 |
| Ubiquitin | Proteintech | Cat# 10201-2-AP, RRID: AB_671515 |
| His-Tag | Proteintech | Cat# 66005-1-Ig, RRID: AB_11232599 |
| Flag-Tag | Proteintech | Cat# 66008-4-Ig, RRID: AB_2918475 |
| HA-Tag | Proteintech | Cat# 66006-2-Ig, RRID: AB_2881490 |
| MMP1 | Proteintech | Cat# 10371-2-AP, RRID: AB_2297741 |
| MMP3 | Proteintech | Cat# 17873-1-AP, RRID: AB_2146587 |
| β‐actin | Proteintech | Cat# 66009-1-Ig, RRID: AB_2687938 |
| GAPDH | Proteintech | Cat# 60004-1-Ig, RRID: AB_2107436 |

**Table S7 sequences of RNA probes**

| Probes | Sequences |
| --- | --- |
| NC | TTGTACTACACAAAAGTACTG |
| Probe#1 | AACTGAAAGCAGACAGGGACTAGATGAACAGTATA |
| Probe#1 | TGAGGGAAGTGTGGACATGGACTAAATATTAAATG |
| m6A probe#1 | AACTGAAAGCAGACAGGGm6ACTAGATGAACAGTATA |
| m6A probe#1 | TGAGGGAAGTGTGGACATGGm6ACTAAATATTAAATG |
